# Genetic association of the tachykinin receptor 1 *TACR1* gene in bipolar disorder, attention deficit hyperactivity disorder, and the alcohol dependence syndrome

**DOI:** 10.1002/ajmg.b.32241

**Published:** 2014-05-09

**Authors:** Sally I Sharp, Andrew McQuillin, Michael Marks, Stephen P Hunt, S Clare Stanford, Greg J Lydall, Marsha Y Morgan, Philip Asherson, David Curtis, Hugh M D Gurling

**Affiliations:** 1 Molecular Psychiatry Laboratory, Division of Psychiatry, University College LondonLondon, UK; 2 UCL Institute for Liver & Digestive Health, Royal Free Campus, University College LondonLondon, UK; 3 Research Department of Neuroscience, Physiology and Pharmacology, Faculty of Life Sciences, University College LondonLondon, UK; 4 ADHD Genetics Group, MRC Social Genetic and Developmental Psychiatry Centre, Institute of Psychiatry, King's College LondonLondon, UK

**Keywords:** tachykinin receptor, mutation, susceptibility, replication, affective disorders, bipolar disorder, alcohol dependence

## Abstract

Single nucleotide polymorphisms (SNPs) in the tachykinin receptor 1 gene (*TACR1*) are nominally associated with bipolar affective disorder (BPAD) in a genome-wide association study and in several case-control samples of BPAD, alcohol dependence syndrome (ADS) and attention-deficit hyperactivity disorder (ADHD). Eighteen *TACR1* SNPs were associated with BPAD in a sample (506 subjects) from University College London (UCL1), the most significant being rs3771829, previously associated with ADHD. To further elucidate the role of *TACR1* in affective disorders, rs3771829 was genotyped in a second BPAD sample of 593 subjects (UCL2), in 997 subjects with ADS, and a subsample of 143 individuals diagnosed with BPAD and comorbid alcohol dependence (BPALC). rs3771829 was associated with BPAD (UCL1 and UCL2 combined: *P* = 2.0 × 10^−3^), ADS (*P* = 2.0 × 10^−3^) and BPALC (*P* = 6.0 × 10^−4^) compared with controls screened for the absence of mental illness and alcohol dependence. DNA sequencing in selected cases of BPAD and ADHD who had inherited *TACR1*-susceptibility haplotypes identified 19 SNPs in the promoter region, 5′ UTR, exons, intron/exon junctions and 3′ UTR of *TACR1* that could increase vulnerability to BPAD, ADS, ADHD, and BPALC. Alternative splicing of *TACR1* excludes intron 4 and exon 5, giving rise to two variants of the neurokinin 1 receptor (NK1R) that differ in binding affinity of substance P by 10-fold. A mutation in intron four, rs1106854, was associated with BPAD, although a regulatory role for rs1106854 is unclear. The association with *TACR1* and BPAD, ADS, and ADHD suggests a shared molecular pathophysiology between these affective disorders. © 2014 The Authors. American Journal of Medical Genetics Part B: Neuropsychiatric Genetics Published by Wiley Periodicals.

## INTRODUCTION

Bipolar affective disorder (BPAD) has a lifetime risk of up to 1.5% [Merikangas et al., 2011]. The genes responsible for BPAD also increase susceptibility to unipolar affective disorder, suicidality, cyclothymia, and hypomania [Bertelsen et al., 1977]. Alcohol dependence syndrome (ADS) is strongly comorbid with BPAD, with 38–50% of bipolar cases also having a diagnosis of an alcohol use disorder [Angst et al., 2006; Goldstein et al., 2006]. In one study, up to 36% of patients with BPAD had a positive family history of alcohol dependence among first-degree relatives [Mantere et al., 2012]. There is also a strong relationship between adolescent attention deficit hyperactivity disorder (ADHD) and adult alcohol dependence [Edwards and Kendler, 2012] with at least 30% of subjects with ADHD reported to develop an alcohol use disorder [Wilens et al., 2011; Tuithof et al., 2012].

Previous genetic studies of bipolar and unipolar affective disorder comorbid alcohol dependence show replicated significant linkage in multiply affected alcoholism families [Dick et al., 2002; Lappalainen et al., 2004; Guerrini et al., 2005]. A genome wide association study (GWAS) of combined alcohol dependence syndrome and bipolar disorder, BPALC, implicated several genes, *CDH11*, *COL11A2*, *NMUR2*, *XPO7*, and *SEMA5A*, which had previously been shown to be associated with ADS [Lydall et al., 2011]. Several genes such as *CDH13*, *CSMD2*, *GRID1*, and *HTR1B* were implicated in susceptibility to unipolar depression comorbid with alcohol dependence [Edwards et al., 2012]. Ten SNPs in the tachykinin receptor 1 (*TACR1*) gene were nominally associated with BPALC, including the intronic marker, rs3771829 (*P* = 3.0 × 10^−3^) [Lydall et al., 2011]. The *TACR1* gene is located on chromosome 2 and encodes the neurokinin 1 receptor which primarily binds the tachykinin, substance P. These tachykinin receptors are G-protein coupled receptors containing seven hydrophobic transmembrane spanning regions [Maggi, 1995]. A synonymous SNP in exon 1 of *TACR1*, rs6715729, has been associated with ADS compared with screened controls in a Caucasian population (*P* = 0.0006, odds ratio (OR) = 6.13, 95% confidence intervals (CI) = 4.06–9.23). The authors also report two risk haplotypes for ADS in the 5′ end of *TACR1*, formed by the three-SNP combinations of rs6715729-rs735668-rs6741029 [Seneviratne et al., 2009]. More recently, five 3′ and 5′ *TACR1* SNPs, rs3771863, rs3755459, rs10490308, rs11688000, and one SNP in a stop codon, rs1106855, were significantly related to ADS severity [Blaine et al., 2013]. Functional magnetic resonance imaging (fMRI) responses to alcohol cues showed three of these genetic markers, which may affect *TACR1* transcription and/or translation, were associated with brain regions in the mesocorticolimbic pathway [Blaine et al., 2013].

Neurokinin 1 receptors (NK1R) encoded by *TACR1* are highly expressed in brain regions associated with reward and reinforcement. The binding density of NK1R is highest in the locus coeruleus, which is important for mood regulation and response to stress [Caberlotto et al., 2003]. Mice with functional ablation of NK1R (*Nk1r*^*−/−*^) have significantly reduced ethanol intake while acute blockade of NK1Rs in wild type mice mimics this effect on alcohol consumption. Inactivation of NK1Rs critically modulates alcohol reward and escalation, supporting a direct role of NK1R in the regulation of alcohol intake [Thorsell et al., 2010], further implicating NK1R function in the development of alcohol dependence. The effects of NK1R antagonism on alcohol and drug reward appear to be selective [Thorsell et al., 2010], involving dopaminergic pathways from the ventral tegmental area of the midbrain to the cerebral cortex and also ascending serotonergic pathways [Commons, 2009]. However, the direct effect of NK1R on mesolimbic dopaminergic signalling remains unclear [Rupniak and Jackson, 1994]. Furthermore, *Nk1r*^*−/−*^ mice are hyperactive and have an atypical response to psychostimulants. They also express greater impulsivity and inattentiveness than wild types in the 5-Choice Serial Reaction-Time Task and are proposed as a model for ADHD [Yan et al., 2009].

To date, allelic associations have been found between five *TACR1* SNPs and BPAD in family-based association studies [Perlis et al., 2008] and several GWAS studies [Ferreira et al., 2008; Sklar et al., 2008]. Within 50 kb of *TACR1*, 18 SNPs out of a total of 80 were significantly associated with BPAD in the UCL1 sample of 506 BPAD subjects, with rs3771829 showing the strongest association (*P* = 2.5 × 10^−3^). A further 10 SNPs were associated with BPAD in the Systematic Treatment Enhancement Protocol for Bipolar Disorder (STEP-BD) and Wellcome Trust Case Control Consortium (WTCCC) samples. When all three samples were combined, seven SNPs were associated with BPAD [Ferreira et al., 2008; Sklar et al., 2008]. The pattern of these SNPs differed in each sample suggesting allelic and haplotypic heterogeneity in disease susceptibility. None of the *TACR1* SNPs were associated with BPAD at the level of genome-wide significance in any one sample although the combined evidence supported the *TACR1* association with BPAD. The top Psychiatric Genetics Consortium (PGC) SNP for ADHD in *TACR1*, located approximately 5 kb downstream, is rs4614953 [Neale et al., 2010], close to the PGC BPAD associated marker, rs2422090 [Sklar et al., 2011]. Linkage disequilibrium (LD) analysis shows that these SNPs are in LD in the European samples of the 1000 genomes project [1000 Genomes Project Consortium, 2010], with an r^2^ of 1.0. Four *TACR1* SNPs, including the top two UCL BPAD SNPs, rs3771829 and rs3771833, which are in LD with one another, were associated with ADHD (*P* = 0.01–0.00008) [Yan et al., 2010]. The two UCL BPAD *TACR1* markers are not in LD with the PGC ADHD and BPAD SNPs, rs4614953 and rs2422090, or the SNP, rs6715729, associated with ADS (data not shown), suggesting that independent genetic risk factors in *TACR1* predict affective disorder phenotypes. The aim of this study is to further investigate the association of *TACR1* with BPAD, BPALC, ADS, and ADHD.

## METHODS

### UCL Clinical Sampling

The UCL BPAD cohort consists of 1,099 individuals. These were sampled in two cohorts. The first cohort (UCL1) comprised 506 bipolar I cases [Ferreira et al., 2008; Sklar et al., 2008] while the second cohort (UCL2) comprised 409 bipolar I (69%) and 184 bipolar II cases [Dedman et al., 2012]. Among the UCL1 BPAD cases were 143 with comorbid ADS according to Research Diagnostic Criteria (RDC) [Lydall et al., 2011]. All UCL bipolar cases were interviewed by a psychiatrist using the lifetime version of the Schedule for Affective Disorders and Schizophrenia-Lifetime Version (SADS-L) schedule18 [Spitzer and Endicott, 1977], rated with the 90-item Operational Criteria Checklist (OPCRIT) [McGuffin et al., 1991] and met diagnostic criteria for bipolar disorder according to RDC [Spitzer et al., 1978]. The UCL ADS sample comprised 997 ADS cases, recruited as part of the UK-COGA (United Kingdom Collaborative Study on the Genetics of Alcoholism) study, were diagnosed using a version of the SSAGA-II questionnaire modified for the UK [Bucholz et al., 1994] and met diagnostic criteria according to DSM-IV and ICD-10. ADS cases were also rated with the OPCRIT. Thirty-five cases of ADHD, diagnosed by experienced clinicians using DSM-IV criteria from two samples, one collected at Cardiff University and the second from the Institute of Psychiatry, London [Yan et al., 2010] were used for DNA sequencing.

The sample of 1,056 normal controls comprised 672 screened controls who were interviewed with the initial clinical screening questions of the SADS-L and selected on the basis of not having a family history of schizophrenia, alcohol dependence or BPAD, for having no past or present personal history of any RDC-defined mental disorder, and were not heavy drinkers; plus 384 unscreened British normal volunteers provided by European Collection of Animal Cell Cultures (ECACC). All cases and controls were selected to be of UK or Irish ancestry as described previously [Datta et al., 2010]. UK National Health Service multicenter and local research ethics approvals were obtained and signed informed consent was given by all subjects. Genomic DNA was obtained from frozen whole blood samples for cases and controls in UCL1 and from saliva samples for the cases in UCL2. DNA was extracted for all samples using methods we have published previously [Pereira et al., 2011] and quantified with PicoGreen (Invitrogen, Paisley, UK) by fluorimetry.

### Sequencing

A total of 32 BPALC subjects from the UCL1 BPAD cohort along with 35 cases of ADHD and a further 32 random normal comparison subjects from the control sample were selected for sequencing, if they had inherited a *TACR1* susceptibility haplotype, based on the criteria of whether an individual was homozygous or heterozygous for the two GWAS *TACR1* SNP markers rs3771829 and rs3771833 alleles. Sequencing was carried out on the promoter region, 1000 base pairs upstream of the transcriptional start site, 5′ untranslated region (UTR), the exons, intron/exon junctions and the entire 3' UTR of *TACR1* isoform 1 (NM_001058.3) which contains all five exons (Table SI). Sequencing was done using the Big Dye terminator v3.1 Cycle Sequencing kit (Applied Biosystems, Warrington, UK) on an ABI 3730*xl* DNA Analyzer (Applied Biosystems). Sequencing data were analysed using the Staden Package [Staden, 1996].

### Genotyping and Association Analysis

To determine whether *TACR1* increases susceptibility to affective disorders, KBiosciences allele-specific PCR (KASPar) (LGC Genomics KBioscience, Hoddesdon, UK) or TaqMan (Applied Biosystems) genotyping assays were designed. The top two *TACR1* UCL1 BPAD GWAS SNPs, rs3771829 and rs3771833, and two SNPs, rs3771856 and rs17011370, also associated with ADHD [Yan et al., 2009], were KASPar genotyped on a LightCycler 480 Real-Time PCR System (Roche, Burgess Hill, UK) in UCL1 and UCL2 BPAD and ADS samples, and screened and unscreened controls. Where *TACR1* nucleotide changes were detected by sequencing the ADHD and BPALC cases, KASPar genotyping was then performed in the UCL1 and UCL2 BPAD samples and controls. Rare variants, potentially aetiological SNPs or SNPs associated with BPAD were genotyped using KASPar in the UCL ADS samples. One SNP, rs1106854, is a triallelic base, therefore two KASPar genotyping assays were carried out, one for each of the minor alleles. For one SNP, rs13387833, a KASPar assay could not be successfully designed and a TaqMan genotyping assay (Applied Biosystems) was carried out in all cases and controls. Quality control to confirm the reproducibility of genotypes was performed as described previously [Dedman et al., 2012]. All these data were analysed to confirm Hardy–Weinberg equilibrium (HWE). Genotypic and allelic associations for SNPs were tested using Fisher's exact, χ^2^ or Cochrane trend tests. Significance values shown for all analyses are uncorrected for multiple testing and a cut-off significance value of *P* < 0.05 was used.

Bioinformatic analysis to determine potentially functional SNPs was carried out using the UCSC genome browser (http://genome.ucsc.edu/), Transcription Element Search System (TESS) [Schug, 2008], Codon Plot (http://www.bioinformatics.org/sms2/codon_plot.html), exonic splicing enhancer prediction server RESCUE-ESE [Fairbrother et al., 2002], Alternative Splice Site Predictor (ASSP) [Wang and Marin, 2006], and MicroInspector (http://bioinfo.uni-plovdiv.bg/microinspector/). 1000 genomes data [1000 Genomes Project Consortium, 2010] was downloaded and imputation analysis was performed using IMPUTE2 [Howie et al.,^2009,2011^] and SNPTEST version 2.0 using the frequentist association test [Marchini and Howie, 2010]. The Ensembl Variant Effect Predictor (VEP) [McLaren et al., 2010] was used to predict the functional consequences of known and unknown variants and regulatory region variants were analysed in the ENCODE data [ENCODE Project Consortium, 2011].

## RESULTS

### TACR1 Association Analysis

In order to investigate whether *TACR1* increases susceptibility to affective disorders we analysed the effect of the top two UCL GWAS SNP markers, rs3771829 and rs3771833, in the combined UCL1 and UCL2 sample of BPAD (Table1). Genotype data did show significant association with BPAD in comparison with screened controls with a confirmed negative history of bipolar disorder and alcohol dependence (rs3771829: *P* = 0.002, OR 1.57, CI 1.18–2.08; rs3771833: *P* = 0.004, OR 1.43, CI 1.12–1.83) but not relative to unscreened controls (Table1). Neither SNP was associated with BPAD in the UCL2 sample alone (data not shown) but both SNPs were associated in UCL1 alone as well as in combination with UCL2. As reported previously [Lydall et al., 2011], one of these SNPs was associated with the sub-group of BPALC cases compared with screened controls (rs3771829: *P* = 0.005, OR 1.87, CI 1.20–2.92) (Table1). Since the association with BPAD may be driven by the subsample of patients with comorbid ADS, the BPALC subgroup was removed from the UCL1 BPAD analysis. A significant association was still observed between BPAD and rs3771829 (*P* = 0.01, OR 1.58, CI 1.11–2.24) and rs3771833 (*P* = 0.04, OR 1.39, CI 1.02–1.90) (Table SII). When we genotyped these SNPs in the UCL ADS sample, rs3771829 was associated with ADS when compared with the screened controls (*P* = 0.002, OR 1.56, CI 1.17–2.09) (Table1). Furthermore, when the UCL BPAD and ADS samples were combined, there was an enhanced significant association with both rs3771829 and rs3771833 when compared with screened controls (*P* = 0.0009, OR 1.57, CI 1.20–2.04; *P* = 0.009, OR 1.36, CI 1.08–1.71, respectively). Since, we were unable to confirm the association between *TACR1* and BPAD, BPALC or ADS with our unscreened controls all subsequent case control analysis has been carried out using the screened control sample.

**I:** Replicated Tests of Association for GWAS *TACR1* SNPs in the UCL1 and UCL2 Bipolar Affective Disorder Cases, Comorbid Bipolar Alcohol Dependence Subsample, and Alcohol Dependence Syndrome Cases Compared With Both Screened and Unscreened Controls

		rs3771833^a^							rs3771829^a^						
	Sample size	MAF^a^	Screened controls^b^		Unscreened controls^c^		Combined controls		MAF^d^	Screened controls^a^		Unscreened controls^c^		Combined controls	
			*P*-value^e^	OR (95 % CI)^f^	*P*-value^e^	OR (95 % CI)^f^	*P*-value^e^	OR (95 % CI)^f^		*P*-value^e^	OR (95% CI)^f^	*P*-value^e^	OR (95% CI)^f^	*P*-value^e^	OR (95% CI)^f^
BPAD^g^	1099	0.118	0.0043	1.43 (1.12–1.83)	0.511	1.09 (0.84–1.42)	0.0189	1.27 (1.04–1.56)	0.092	0.0018	1.57 (1.18–2.08)	0.657	0.94 (0.71–1.24)	0.057	1.24 (0.99–1.56)
BPALC^h^	143	0.120	0.0759	1.45 (0.96–2.20)	0.608	1.12 (0.73–1.71)	0.572	1.17 (0.74–1.70)	0.108	0.0051	1.87 (1.20–2.92)	0.615	1.12 (0.72–1.75)	0.057	1.49 (0.99–2.24)
ADS^i^	997	0.107	0.0537	1.28 (1.00–1.65)	0.878	0.98 (0.75–1.28)	0.216	1.14 (0.93–1.41)	0.092	0.0022	1.56 (1.17–2.09)	0.649	0.94 (0.70–1.25)	0.066	1.24 (0.99–1.56)
BPAD + ADS	2,096	0.113	0.0087	1.36 (1.08–1.71)	0.769	1.04 (0.81–1.33)	0.150	1.16 (0.95–1.42)	0.092	0.0009	1.57 (1.20–2.04)	0.628	0.94 (0.72–1.22)	0.034	1.27 (1.02–1.59)
Screened controls	672	0.086	—	—	0.086	1.31 (0.96–1.79)	—	—	0.061	—	—	0.003	1.67 (1.19–2.36)	—	—
Unscreened controls	384	0.109	—	—	—	—	—	—	0.098	—	—	—	—	—	—

a dbSNPrsID, Reference single nucleotide polymorphism (rs) ID given. Position on chromosome 2; hg19 Build NCBI37, May 2009, associated gene, NM_001058: rs3771833 (T > C) 75,366,937; rs3771829 (G > C) 75,364,145.

b Screened control population has been screened for a history of mental illness and drinking behavior.

c Unscreened control population has not been screened for a history of mental illness and drinking behavior.

d MAF, minor allele frequency.

e *P*-value.

f OR(95 % CI), odds ratio with 95% confidence intervals in parentheses.

g BPAD bipolar affective disorder University College London sample numbers 1 and 2 (UCL1 & UCL2) combined.

h BPALC, subsample of UCL1 BPAD with comorbid alcohol dependence syndrome.

i ADS, alcohol dependence syndrome University College London sample.

### Detection and Evaluation of Other Variants in TACR1

A total of 19 SNPs were detected by sequence analysis across the promoter region, 5′ UTR, exons, intron/exon junctions and 3′ UTR of *TACR1*, of which one was novel (Table SIII). These included one synonymous coding base pair change, rs6715729; nine promoter SNPs: rs59099335, rs34374747, rs1477157, rs1477156, rs13387833, rs2111375, rs2193405, rs13384011, and rs10210648; one SNP in the exon 1 5′ UTR, rs200655774; five intronic SNPs: one in intron 1, rs2024512, one in intron 3, rs78052302, and three in intron 4, rs201914096, rs1106854, and rs1106855 (not genotyped); and five SNPs in the 3′ UTR of exon 5: rs881, ss825678898, rs17010664, rs62148938, and rs12713828.

Bioinformatic analysis of the promoter region SNPs for altered transcription factor binding sites indicated that the mutant alleles of all promoter and 5-UTR SNPs are likely to both introduce new transcription factor binding sites and prevent binding of some transcription factors compared to their respective common alleles (TESS). The Mfold program showed that the 5′ UTR rs200655774 base pair change is unlikely to significantly alter the secondary structure of *TACR1* mRNA. The minor allele of the exon 1 synonymous SNP, rs6715729, results in a modest reduction in codon usage (Phe TTT 57% >Phe TTC 43% frequency, Codon Plot) but is not predicted to be an exonic splicing enhancer (RESCUE-ESE). The five 3-UTR SNPs are all predicted to gain and/or lose miRNA binding sites (MicroInspector). One intronic SNP, rs201914096, is predicted to introduce an alternative isoform/cryptic splice site acceptor with a splice site strength of 5.676, which has a greater than 95% likelihood of being a functional splice site (ASSP) [Wang and Marin, 2006]. The only SNP found by sequencing to be associated in the combined UCL1 and UCL2 BPAD sample compared to screened controls (Table SIII), the intronic triallelic base rs1106854, does not alter a splice site (ASSP). An additional SNP, rs17011370, previously associated with ADHD [Yan et al., 2010] is nominally associated with the BPALC clinical subgroup (Table SIV). Six SNPs were genotyped in ADS because they had either been associated with ADHD previously [Yan et al., 2010], or there was an increased frequency in sequenced cases compared to sequenced controls, or based on predicted functional effects. None of these were associated with ADS (Table SV).

### Genotype Analysis of Screened Controls Versus Unscreened Controls

Significant differences in allele frequencies were observed between the screened and unscreened controls (Table SVI). In particular, the rs3771829 allele frequency was significantly different between screened and unscreened controls (*P* = 0.003, OR 1.67, CI 1.19–2.36). It is interesting that the unscreened controls have similar allele frequencies to those of the 1000 genome controls and the WTCCC controls (data not shown).

### Imputed Tests of Association in TACR1 in Bipolar Disorder and in Comborbid Bipolar Alcohol Dependence

Imputation analysis using IMPUTE2 and SNPTEST predicted that several regulatory region SNPs, as well as variants located both upstream, downstream and in introns of *TACR1* are significantly associated in the UCL1 and UCL2 BPAD samples (Table SVII) and in the BPALC subgroup (Table SVIII). Two synonymous variants in *TACR1* were imputed to be associated with BPALC. In exon 5, rs34117315 results in a modest reduction in codon usage (Ser TCG 15% > Ser TCA 13% frequency, Codon Plot). The second variant in exon 3, chr2:75280825, also reduces codon usage (Ser TAT 58% > Ser TAC 42%, Codon Plot). Neither variant was predicted to be an exonic splicing enhancer (RESCUE-ESE). Several imputed regulatory region variants were predicted to be in regions showing enrichment for the H3K27Ac histone mark, which is the acetylation of lysine 27 of the H3 histone protein, often found near active regulatory elements (ENCODE).

## DISCUSSION

Genetic association with *TACR1* and BPAD was not found in the UCL2 replication cohort for the markers most strongly associated in the UCL1 GWAS sample [Sklar et al., 2008]. This result is common in the field of complex genetic diseases reflecting both the heterogeneity for bipolar disorder susceptibility genes, even within a single ancestrally selected group of cases and controls, and the presence of low frequency disease alleles. The association with the top two GWAS hits held when the UCL1 and UCL2 BPAD samples were combined. We also report replicated significant association with intron 1 *TACR1* mutations in BPAD in the BPALC subgroup and ADS cases in comparison with a screened population of controls.

Sequencing of *TACR1* in BPALC and ADHD cases detected one novel base pair change in the 3′ UTR, although this was not significantly associated with BPAD when compared to screened controls. Genotyping of an additional 18 database SNPs found by sequencing *TACR1* identified only one marker, rs1106854, positively associated with BPAD. Any possible regulatory role for this intron 4 variant is unclear. The *TACR1* gene is alternatively spliced to exclude intron 4 and exon 5 of the gene, which gives rise to two naturally occurring variants of NK1R. Truncated NK1R lacks 96 amino acid residues corresponding to the C-terminus of the full length receptor. Furthermore, activation of full length and truncated NK1R results in differential receptor signalling mediated by different G-proteins [Tuluc et al., 2009] and the truncated form has a 10-fold lower binding affinity to substance P than the long form [Fong et al., 1992]. The long NK1R isoform is prevalent throughout the human brain, while the truncated form is more common in peripheral tissues, but to date there is little evidence for a region-specific role for the two isoforms in the CNS [Caberlotto et al., 2003]. Other regions of the *TACR1* gene still need to be screened for mutations: for example, the whole of intron 4 and splice sites responsible for the alternative splicing of *TACR1*. We did not identify any other splice site SNPs that would result in differential expression of the two *TACR1* isoforms in intron 4 in UCL1 and UCL2 BPAD cases, but the association with rs1106854 warrants further investigation. From the BPALC sub-analysis, there was significant association with the intergenic SNP, rs17011370 located approximately 270 kb upstream of *TACR1*.

The association between intronic loci in both BPAD and ADS relative to screened controls supports previous evidence of association in ADHD and further implicates a role for *TACR1* as both a functional and positional candidate gene with the potential to increase susceptibility to alcohol dependence and affective disorders. We did not find a significant association with controls who had not been screened for a history of mental illness or drinking behavior. These data highlight the importance of using the appropriate control group and to know the level of drinking in a control population, as well as family histories of psychiatric diagnoses, for true genetic associations to be assessed [Nelson et al., 2013]. It is also possible that the differences we observe between ADS cases and controls are due to population stratification. While there was a significant difference between BPAD in the absence of comorbid alcohol dependence and screened controls for the two top GWAS hits, the association was much stronger in the BPALC subset relative to screened controls. Thus, it is likely to be the comorbid ADS present in a subsample of the BPAD cohort that is driving the association we observe with BPAD and not the absence of drinking behavior in the screened controls. Our data provide further evidence of an association between *TACR1* and ADS as found previously [Seneviratne et al., 2009]. We did not replicate the significant association with rs6715729 reported by Seneviratne et al. [2009] in the UCL ADS sample, but a more recent study highlighted several other *TACR1* variants that predict fMRI responses to alcohol cues and alcohol dependence [Blaine et al., 2013]. From our imputation analysis, only two of the five SNPs reported in the study by Blaine et al. [2013] were imputed from our data, but neither SNP was significantly associated with either BPAD or BPALC.

The NK1R is an attractive molecular target for the treatment of depression and anxiety [Ebner et al., 2009]. Previous in vivo studies show that *Nk1r*^*−/−*^ mice display increased alcohol drinking behavior [Thorsell et al., 2010] and NK1R antagonist treatment significantly inhibits operant self-administration of 10% ethanol compared with vehicle in rats [Steensland et al., 2010; Schank et al., 2013]. Interestingly, a SNP upstream of *TACR1* present in alcohol-preferring rats increased transcription factor binding, gene transcription, alcohol self-administration and sensitivity to the NK1R antagonist L822429 [Schank et al., 2013]. In a randomized controlled study in recently detoxified in-patients with ADS, the NK1R antagonist, LY686017, suppressed alcohol cravings. Brain fMRI responses to affective stimuli likewise suggested beneficial effects for the treatment of ADS [George et al., 2008]. The early results in treating affective disorder with the NK1R antagonist aprepitant were promising, but no effect was found in a controlled treatment trial of depression [Hafizi et al., 2007; McCabe et al., 2009; Chandra et al., 2010]. It is possible that only a small genetic subgroup of ADS, ADHD and BPAD cases would benefit from aprepitant, which points to a personalised targeting of this drug based on genetic findings. So far the intronic SNP rs3771829 shows the greatest promise as a biomarker for prediction of treatment effects from NK1R antagonists.

Taking our results together, we conclude that polymorphisms in *TACR1* significantly increase susceptibility to BPAD, ADS, as well as ADHD. The significant *TACR1* allele frequency difference between our screened and unscreened controls also suggests an effect from *TACR1* on normal drinking behavior. Additional studies are needed to replicate these results in other samples with access to screened and unscreened controls and to elucidate the regulatory mechanism(s) by which these polymorphisms affect NK1R function in the brain.
