# Supplementary material for: Genetic association of the tachykinin receptor 1 TACR1 gene in bipolar disorder, attention deficit hyperactivity disorder, and the alcohol dependence syndrome
Source: Am J Med Genet B Neuropsychiatr Genet. 2014 May 9;165(4):373–80. doi: 10.1002/ajmg.b.32241 (PMC4278563; doi:10.1002/ajmg.b.32241)
Supplement: Supplementary file 1 — Supporting Information. [file ajmg0165-0373-sd1.docx]

**eTable I. *TACR1* PCR sequencing primers.**

| Primer name | Sequence 5’ - 3’ | Primer position on Chr 2 (bp)^1^ |
| --- | --- | --- |
| TACR1_PRM1_F | ATTTGAGCCCTCCACCTGAC | 75427314 |
| TACR1_PRM1_R | GAGTCCCCGCAGTGAAAG | 75427768 |
| TACR1_PRM2_F | GAGAAGCCAAGATGGGATGA | 75427888 |
| TACR1_PRM2_R | CAGCTCTTTGAGATGGTGGA | 75428328 |
| TACR1_PRM3_F | CAAGAGAACAGCGTCCCTTT | 75428233 |
| TACR1_PRM3_R | GTTAGGTGGCCTCCCTCTTC | 75428795 |
| TACR1_PRM4_F | GGGGGTGAACAAGCACTTAC | 75428686 |
| TACR1_PRM4_R | CAGAGTTGAAGGCCAGGGTA | 75429208 |
| TACR1_PRM5_F | TAGCCCTCGGCTAACTTTGA | 75429116 |
| TACR1_PRM5_R | TCCTGTGTTGAGTGTCAAGGA | 75429609 |
| TACR1_PRM6_F | TGAATTTTCCCCCTACAGCTT | 75429480 |
| TACR1_PRM6_R | CTTCCCAGCAATTCCACTTT | 75430009 |
| TACR1_1_F | GCAGCTGGAATCGTCGTTGC | 75426908 |
| TACR1_1_R | TGGCTCCTGCGCTCACCA | 75427389 |
| TACR1_2_F | GGCCAGGATTCTGGAGCTTCG | 75426537 |
| TACR1_2_R | GTCGCCTCTGGCTTCTCGGT | 75427110 |
| TACR1_3_F | ACGCTTCTGGATGCACTGCC | 75426229 |
| TACR1_3_R | CGGCGGTGGAATGAGACAGTT | 75426770 |
| TACR1_4_F | CGTTGTGGACAGCATAGGTGAAGT | 75425772 |
| TACR1_4_R | GACCGGTGGAATTTCTTTCCCT | 75426350 |
| TACR1_5_F | TTGAAAGGAAGAGAGAGAGTGATGGG | 75425453 |
| TACR1_5_R | CCTCCCGGTGGACTCAGACCT | 75426029 |
| TACR1_6_F | CCTGACAACTGTGGACATATCAACCTG | 75347527 |
| TACR1_6_R | CGCATGTTGGCATTGTATGGC | 75348066 |
| TACR1_7_F | GTGGCTCATGCCTGTCCAGC | 75280540 |
| TACR1_7_R | GACAAGTCTCCCTGGAGTGGGC | 75281019 |
| TACR1_8_F | ATCAGTCCACTCCGGGCTCC | 75278182 |
| TACR1_8_R | GAAGCAGCCGTGAGAGAGGG | 75278615 |
| TACR1_9_F | TTTCTGCATGCATAGCCCGC | 75277965 |
| TACR1_9_R | CTGGCCATGAGCTCCACCAT | 75278414 |
| TACR1_10_F | TCTGATGGTTCCAGATGAAGGGA | 75276470 |
| TACR1_10_R | TTGGCCTCAGCATCTCCAGC | 75276984 |
| TACR1_11_F | GGGACATAATGTGGAAGCCCG | 75275939 |
| TACR1_11_R | TGCCTTTGACCTGCCTCCCT | 75276507 |

^1^ GRCh37/hg19 Assembly

**eTable II: Tests of association with *TACR1* SNPs in UCL bipolar disorder samples without alcohol dependence relative to screened controls**

| **SNP ID ^1^** | **Position Chr 2 (NCBI37/hg19)^2^** | **BPnoALC*vs*. Screened Controls^3^** | **N**^4^ | **Minor Allele Frequency** | **Genotype counts** | ***P* Value**^5^ | **OR (95 % CI)**^6^ |
| --- | --- | --- | --- | --- | --- | --- | --- |
| rs17011370 | 75,692,623 | Case | 349 | 0.05 | TT 0; CT 34; CC 315 | 0.16 | 0.74 (0.49 - 1.13) |
|  |  | Control | 549 | 0.07 | TT 2; CT 67; CC 480 |  |  |
| rs59099335 | 75,429,888 | Case | 358 | 0.08 | TT 1; CT 53; CC 304 | 0.74 | 0.94 (0.66 - 1.34) |
|  |  | Control | 549 | 0.08 | TT 6; CT 77; CC 466 |  |  |
| rs34374747 | 75,429,380 | Case | 350 | 0.25 | GG 21; AG 131; AA 198 | 0.66 | 0.95 (0.76 - 1.19) |
|  |  | Control | 532 | 0.26 | GG 47; AG 179; AA 306 |  |  |
| rs1477157 | 75,429,228 | Case | 359 | 0.50 | AA 89; AG 184; GG 86 | 0.75 | 0.97 (0.80 - 1.17) |
|  |  | Control | 557 | 0.50 | AA 142; AG 269; GG 146 |  |  |
| rs1477156 | 75,429,141 | Case | 358 | 0.51 | TT 82; CT 188; CC 88 | 0.80 | 0.98 (0.81 - 1.18) |
|  |  | Control | 559 | 0.51 | TT 134; CT 275; CC 150 |  |  |
| rs13387833 | 75,428,396 | Case | 361 | 0.09 | TT 6; CT 51; CC 304 | 0.71 | 0.94 (0.68 - 1.30) |
|  |  | Control | 590 | 0.09 | TT 2; CT 105; CC 483 |  |  |
| rs2111375 | 75,427,878 | Case | 357 | 0.27 | AA 23; AG 146; GG 188 | 0.49 | 0.93 (0.75 - 1.15) |
|  |  | Control | 564 | 0.29 | AA 55; AG 210; GG 299 |  |  |
| rs13384011 | 75,427,244 | Case | 360 | 0.09 | AA 6; AG 52; GG 302 | 0.62 | 0.92 (0.67 - 1.28) |
|  |  | Control | 559 | 0.10 | AA 2; AG 103; GG 454 |  |  |
| rs2193405 | 75,426,968 | Case | 362 | 0.27 | AA 23; AT 147; TT 192 | 0.69 | 0.96 (0.78 - 1.18) |
|  |  | Control | 560 | 0.28 | AA 51; AT 206; TT 303 |  |  |
| rs10210648 | 75,426,903 | Case | 360 | 0.09 | CC 6; CG 54; GG 300 | 0.81 | 0.96 (0.70 - 1.33) |
|  |  | Control | 563 | 0.10 | CC 2; CG 103; GG 458 |  |  |
| rs200655774 | 75,426,103 | Case | 361 | 0.003 | CC 0; CG 2; GG 359 | 0.66 | 1.54 (0.22 - 10.99) |
|  |  | Control | 557 | 0.002 | CC 0; CG 2; GG 555 |  |  |
| rs6715729 | 75,425,728 | Case | 357 | 0.50 | GG 89; AG 179; AA 89 | 0.77 | 0.97 (0.81 - 1.17) |
|  |  | Control | 54 | 0.51 | GG 150; AG 272; AA 142 |  |  |
| rs2024512 | 75,425,623 | Case | 361 | 0.49 | AA 86; AG 179; GG 96 | 0.93 | 0.99 (0.82 - 1.20) |
|  |  | Control | 559 | 0.49 | AA 137; AG 272; GG 150 |  |  |
| **SNP ID ^1^** | **Position Chr 2 (NCBI37/hg19)^2^** | **BPnoALC *vs*. Screened Controls^3^** | **N**^4^ | **Minor Allele Frequency** | **Genotype counts** | ***P* Value**^5^ | **OR (95 % CI)**^6^ |
| rs3771856 | 75,414,014 | Case | 355 | 0.47 | GG 78; AG 179; AA 98 | 0.59 | 0.95 (0.79 - 1.15) |
|  |  | Control | 562 | 0.49 | GG 134; AG 277; AA 151 |  |  |
| rs3771833 | 75,366,937 | Case | 360 | 0.12 | TT 5; CT 73; CC 282 | 0.036 | 1.39 (1.02 - 1.90) |
|  |  | Control | 561 | 0.09 | TT 6; CT 84; CC 471 |  |  |
| rs3771829 | 75,364,145 | Case | 361 | 0.09 | GG 3; CG 61; CC 297 | 0.010 | 1.58 (1.11 - 2.24) |
|  |  | Control | 566 | 0.06 | GG 2; CG 65; CC 499 |  |  |
| rs78052302 | 75,280,721 | Case | 362 | 0.06 | -- 1; -T 39; TT 322 | 0.15 | 1.37 (0.89 - 2.11) |
|  |  | Control | 560 | 0.04 | -- 3; -T 41; TT 516 |  |  |
| rs201914096 | 75,278,195 | Case | 360 | 0.0014 | TT 0; CT 1; CC 359 | 0.76 | 1.53 (0.10 - 24.57) |
|  |  | Control | 552 | 0.0009 | TT 0; CT 1; CC 551 |  |  |
| rs1106854 | 75,278,014 | Case | 362 | G 0.02, T 0.26 | GG 0; TG 6; CG 12; TT 34; CT 117; CC 193 | 0.21^7^ | − |
|  |  | Control | 555 | G 0.03, T 0.29 | GG 0; TG 4; CG 27; TT 49; CT 222; CC 253 |  |  |
| rs881 | 75,276,429 | Case | 361 | 0.18 | CC 7; CG 115; GG 239 | 0.73 | 1.04 (0.82 - 1.34) |
|  |  | Control | 554 | 0.17 | CC 15; CG 161; GG 378 |  |  |
| ss825678898 | 75,276,401 | Case | 361 | 0.003 | TT 0; -T 2; -- 359 | 0.29 | 0.44 (0.09 - 2.12) |
|  |  | Control | 556 | 0.006 | TT 0; -T 7; -- 549 |  |  |
| rs17010664 | 75,276,290 | Case | 360 | 0.09 | AA 4; AG 58; GG 298 | 0.48 | 0.89 (0.65 - 1.23) |
|  |  | Control | 555 | 0.10 | AA 9; AG 95; GG 451 |  |  |
| rs12713828 | 75,276,081 | Case | 361 | 0.46 | GG 80; AG 171; AA 110 | 0.18 | 1.14 (0.94 - 1.38) |
|  |  | Control | 556 | 0.43 | GG 95; AG 284; AA 177 |  |  |

^1^ SNP, single nucleotide polymorphism. Reference SNP (rs) ID given where available; otherwise Chromosome number_position stated.

^2^ Base position on chromosome 2, hg19 Build NCBI37 May 2009.

^3^ University College London (UCL) bipolar affective disorder (BPAD) 1 subsample of individuals without comorbid alcohol dependence. Screened Controls have been screened for a history of mental illness and alcohol use disorders.

^4^ N, number of samples.

^5^ P Value, Probability value determined with Chi-Square analysis unless otherwise specified.

^6^ OR (95 % CI), odds ratio with 95 % confidence intervals in parentheses.

^7^ rs1106854 is triallelic, Cochrane Armitage Test of Trend (Pearson Chi-Square 9.264, Linear by linear association 1.584, df = 4, N = 917).

**eTable III: Tests of association with *TACR1* SNPs in UCL1 and UCL2 bipolar affective disorder cases relative to screened controls**

| **SNP ID**^1^ | **Position Chr2 (NCBI37/ hg19)**^2^ | **BPAD *vs.* Screened Controls**^3^ | **N**^4^ | **Minor Allele Frequency** | **Genotype counts** | ***P* Value**^5^ | **OR (95 % CI)**^6^ |
| --- | --- | --- | --- | --- | --- | --- | --- |
| rs17011370 | 75,692,623 | Case | 1055 | 0.06 | TT 1; CT 114; CC 940 | 0.27 | 0.84 (0.62 - 1.14) |
|  |  | Control | 549 | 0.06 | TT 2; CT 67; CC 480 |  |  |
| rs59099335 | 75,429,888 | Case | 1054 | 0.07 | TT 4; CT 133; CC 917 | 0.14 | 0.81 (0.62 - 1.07) |
|  |  | Control | 549 | 0.08 | TT 6; CT 77; CC 466 |  |  |
| rs34374747 | 75,429,380 | Case | 1021 | 0.25 | GG 77; AG 361; AA 583 | 0.79 | 0.98 (0.82 - 1.16) |
|  |  | Control | 532 | 0.26 | GG 47; AG 179; AA 306 |  |  |
| rs1477157 | 75,429,228 | Case | 1069 | 0.48 | AA 295; AG 523; GG 251 | 0.19 | 0.91 (0.79 - 1.05) |
|  |  | Control | 557 | 0.50 | AA 142; AG 269; GG 146 |  |  |
| rs1477156 | 75,429,141 | Case | 1068 | 0.49 | TT 283; CT 527; CC 258 | 0.16 | 0.90 (0.78 - 1.04) |
|  |  | Control | 559 | 0.51 | TT 134; CT 275; CC 150 |  |  |
| rs13387833 | 75,428,396 | Case | 1075 | 0.09 | TT 11; CT 174; CC 890 | 0.91 | 0.99 (0.77 - 1.26) |
|  |  | Control | 590 | 0.09 | TT 2; CT 105; CC 483 |  |  |
| rs2111375 | 75,427,878 | Case | 1060 | 0.29 | AA 82; AG 446; GG 532 | 0.81 | 1.02 (0.87 - 1.20) |
|  |  | Control | 564 | 0.28 | AA 55; AG 210; GG 299 |  |  |
| rs13384011 | 75,427,244 | Case | 1062 | 0.09 | AA 10; AG 171; GG 881 | 0.59 | 0.93 (0.73 - 1.20) |
|  |  | Control | 559 | 0.10 | AA 2; AG 103; GG 454 |  |  |
| rs2193405 | 75,426,968 | Case | 1073 | 0.27 | AA 82; AT 411; TT 580 | 0.67 | 0.96 (0.82 - 1.14) |
|  |  | Control | 560 | 0.28 | AA 51; AT 206; TT 303 |  |  |
| rs10210648 | 75,426,903 | Case | 1071 | 0.09 | CC 10; CG 177; GG 884 | 0.78 | 0.96 (0.75 - 1.24) |
|  |  | Control | 563 | 0.10 | CC 2; CG 103; GG 458 |  |  |
| rs200655774 | 75,426,103 | Case | 1073 | 0.0009 | CC 0; CG 2; GG 1071 | 0.50 | 0.52 (0.07 - 3.69) |
|  |  | Control | 557 | 0.0018 | CC 0; CG 2; GG 555 |  |  |
| rs6715729 | 75,425,728 | Case | 1065 | 0.48 | GG 255; AG 515; AA 295 | 0.16 | 0.90 (0.78 - 1.04) |
|  |  | Control | 564 | 0.51 | GG 150; AG 272; AA 142 |  |  |
| rs2024512 | 75,425,623 | Case | 1072 | 0.47 | AA 241; AG 517; GG 314 | 0.22 | 0.91 (0.79 - 1.06) |
|  |  | Control | 559 | 0.49 | AA 137; AG 272; GG 150 |  |  |
| **SNP ID**^1^ | **Position Chr2 (NCBI37/ hg19)**^2^ | **BPAD *vs.* Screened Controls**^3^ | **N**^4^ | **Minor Allele Frequency** | **Genotype counts** | ***P* Value**^5^ | **OR (95 % CI)**^6^ |
| rs3771856 | 75,414,014 | Case | 1072 | 0.46 | GG 234; AG 516; AA 322 | 0.16 | 0.90 (0.78 - 1.04) |
|  |  | Control | 562 | 0.48 | GG 134; AG 277; AA 151 |  |  |
| rs3771833 | 75,366,937 | Case | 1067 | 0.12 | TT 16; TC 220; CC 831 | 0.0043 | 1.43 (1.12 - 1.83) |
|  |  | Control | 561 | 0.09 | TT 6; TC 84; CC 471 |  |  |
| rs3771829 | 75,364,145 | Case | 1072 | 0.09 | GG 8; CG 182; CC 882 | 0.0018 | 1.57 (1.18 - 2.08) |
|  |  | Control | 566 | 0.06 | GG 2; CG 65; CC 499 |  |  |
| rs78052302 | 75,280,721 | Case | 1070 | 0.05 | -- 3; -T 111; TT 956 | 0.12 | 1.32 (0.93 - 1.87) |
|  |  | Control | 560 | 0.04 | -- 3; -T 41; TT 516 |  |  |
| rs201914096 | 75,278,195 | Case | 1067 | 0.0009 | TT 0; CT 2; CC 1065 | 0.98 | 1.04 (0.09 - 11.42) |
|  |  | Control | 552 | 0.0009 | TT 0; CT 1; CC 551 |  |  |
| rs1106854 | 75,278,014 | Case | 1073 | G 0.02, T 0.26 | GG 1; TG 10; CG 35; TT 70; CT 413; CC 544 | 0.03^7^ | – |
|  |  | Control | 555 | G 0.03, T 0.29 | GG 0; TG 4; CG 27; TT 49; CT 222; CC 253 |  |  |
| rs881 | 75,276,429 | Case | 1073 | 0.17 | CC 32; CG 305; GG 736 | 0.98 | 1.00 (0.82 - 1.21) |
|  |  | Control | 554 | 0.17 | CC 15; CG 161; GG 378 |  |  |
| ss825678898 | 75,276,401 | Case | 1070 | 0.004 | -- 0; -T 9; TT 1061 | 0.42 | 0.67 (0.25 - 1.80) |
|  |  | Control | 556 | 0.006 | -- 0; -T 7; TT 549 |  |  |
| rs17010664 | 75,276,290 | Case | 1067 | 0.10 | GG 12; AG 199; AA 856 | 0.81 | 1.03 (0.81 - 1.31) |
|  |  | Control | 555 | 0.10 | GG 9; AG 95; AA 451 |  |  |
| rs12713828 | 75,276,081 | Case | 1068 | 0.45 | GG 222; AG 520; AA 326 | 0.17 | 1.11 (0.96 - 1.28) |
|  |  | Control | 556 | 0.43 | GG 95; AG 284; AA 177 |  |  |

^1^ SNP, single nucleotide polymorphism. Reference SNP (rs) ID given where available; otherwise Chromosome number_position stated.

^2^ Base position on chromosome 2, hg19 Build NCBI37 May 2009.

^3^ University College London (UCL) bipolar affective disorder (BPAD) samples 1 and 2 combined. Note that allele counts for UCL1 are those obtained from our GWAS study (Sklar et al., 2008) and do not overlap with the WTCCC or GSK GWAS datasets. Additional genotyping was performed in the UCL1 samples after the GWAS study and this data has been added to allele counts for non-GWAS SNPs. Controls have been screened for a history of mental illness and alcohol use disorders.

^4^ N, number of samples.

^5^ P Value, Probability value determined with Chi-Square analysis unless otherwise specified.

^6^ OR (95 % CI), odds ratio with 95 % confidence intervals in parentheses.

^7^ rs1106854 is triallelic, Cochrane Armitage Test of Trend (Pearson Chi-Square 8.002, Linear by linear association 4.798, df = 5, N = 1628).

**eTable IV: Tests of association with *TACR1* SNPs in comorbid bipolar disorder and alcohol dependence cases relative to screened controls**

| **SNP ID**^1^ | **Position Chr2 (NCBI37/ hg19)**^2^ | **BPALC *vs.* Screened Controls**^3^ | **N**^4^ | **Minor Allele Frequency** | **Genotype counts** | ***P* Value**^5^ | **OR (95 % CI)**^6^ |
| --- | --- | --- | --- | --- | --- | --- | --- |
| rs17011370 | 75,692,623 | Case | 141 | 0.03 | TT 0; CT 9; CC 132 | 0.04 | 0.48 (0.24 - 0.97) |
|  |  | Control | 549 | 0.07 | TT 2; CT 67; CC 480 |  |  |
| rs59099335 | 75,429,888 | Case | 143 | 0.07 | TT 0; CT 20; CC 123 | 0.53 | 0.85 (0.52 - 1.41) |
|  |  | Control | 549 | 0.08 | TT 6; CT 77; CC 466 |  |  |
| rs34374747 | 75,429,380 | Case | 136 | 0.26 | GG 11; AG 48; AA 77 | 0.60 | 1.00 (0.74 - 1.36) |
|  |  | Control | 532 | 0.26 | GG 47; AG 179; AA 306 |  |  |
| rs1477157 | 75,429,228 | Case | 141 | 0.49 | AA 38; AG 67; GG 36 | 0.75 | 0.96 (0.74 - 1.24) |
|  |  | Control | 557 | 0.50 | AA 142; AG 269; GG 146 |  |  |
| rs1477156 | 75,429,141 | Case | 141 | 0.49 | TT 38; CT 67; CC 36 | 0.52 | 0.92 (0.71 - 1.19) |
|  |  | Control | 559 | 0.51 | TT 134; CT 275; CC 150 |  |  |
| rs13387833 | 75,428,396 | Case | 143 | 0.08 | TT 1; CT 22; CC 120 | 0.66 | 0.90 (0.57 - 1.43) |
|  |  | Control | 590 | 0.09 | TT 2; CT 105; CC 483 |  |  |
| rs2111375 | 75,427,878 | Case | 136 | 0.29 | AA 10; AG 59; GG 67 | 0.82 | 1.03 (0.77 - 1.38) |
|  |  | Control | 564 | 0.28 | AA 55; AG 210; GG 299 |  |  |
| rs13384011 | 75,427,244 | Case | 143 | 0.08 | AA 1; AG 22; GG 120 | 0.54 | 0.87 (0.54 - 1.38) |
|  |  | Control | 559 | 0.10 | AA 2; AG 103; GG 454 |  |  |
| rs2193405 | 75,426,968 | Case | 143 | 0.28 | AA 12; AT 57; TT 74 | 0.78 | 1.04 (0.78 - 1.39) |
|  |  | Control | 560 | 0.28 | AA 51; AT 206; TT 303 |  |  |
| rs10210648 | 75,426,903 | Case | 142 | 0.09 | CC 1; CG 22; GG 119 | 0.59 | 0.88 (0.55 - 1.40) |
|  |  | Control | 563 | 0.10 | CC 2; CG 103; GG 458 |  |  |
| rs200655774 | 75,426,103 | Case | 142 | – | CC 0; CG 0; GG 142 | 0.48 | – |
|  |  | Control | 557 | 0.002 | CC 0; CG 2; GG 555 |  |  |
| rs6715729 | 75,425,728 | Case | 142 | 0.49 | GG 36; AG 68; AA 38 | 0.67 | 0.95 (0.73 - 1.23) |
|  |  | Control | 564 | 0.51 | GG 150; AG 272; AA 142 |  |  |
| rs2024512 | 75,425,623 | Case | 142 | 0.49 | AA 35; AG 68; GG 39 | 0.94 | 0.99 (0.76 - 1.29) |
|  |  | Control | 559 | 0.49 | AA 137; AG 272; GG 150 |  |  |
| **SNP ID**^1^ | **Position Chr2 (NCBI37/ hg19)**^2^ | **BPALC *vs.* Screened Controls**^3^ | **N**^4^ | **Minor Allele Frequency** | **Genotype counts** | ***P* Value**^5^ | **OR (95 % CI)**^6^ |
| rs3771856 | 75,414,014 | Case | 141 | 0.47 | GG 31; AG 71; AA 39 | 0.69 | 0.95 (0.73 - 1.23) |
|  |  | Control | 562 | 0.48 | GG 134; AG 277; AA 151 |  |  |
| rs3771833 | 75,366,937 | Case | 142 | 0.12 | TT 3; TC 28; CC 111 | 0.08 | 1.45 (0.96 - 2.20) |
|  |  | Control | 561 | 0.09 | TT 6; TC 84; CC 471 |  |  |
| rs3771829^7^ | 75,364,145 | Case | 143 | 0.11 | GG 1; CG 29; CC 113 | 0.005 | 1.87 (1.20 - 2.92) |
|  |  | Control | 566 | 0.06 | GG 2; CG 65; CC 499 |  |  |
| rs78052302 | 75,280,721 | Case | 143 | 0.04 | -- 2; -T 8; TT 133 | 1.00 | 1.00 (0.52 - 1.91) |
|  |  | Control | 560 | 0.04 | -- 3; -T 41; TT 516 |  |  |
| rs201914096 | 75,278,195 | Case | 143 | 0.0035 | TT 0; CT 1; CC 142 | 0.30 | 3.87 (0.24 - 62.06) |
|  |  | Control | 552 | 0.0009 | TT 0; CT 1; CC 551 |  |  |
| rs1106854 | 75,278,014 | Case | 143 | G 0.02, T 0.27 | GG 0; TG 0; CG 6; TT 11; CT 55; CC 71 | 0.27^8^ | – |
|  |  | Control | 555 | G 0.03, T 0.29 | GG 0; TG 4; CG 27; TT 49; CT 222; CC 253 |  |  |
| rs881 | 75,276,429 | Case | 142 | 0.16 | CC 8; CG 30; GG 104 | 0.68 | 0.93 (0.65 - 1.32) |
|  |  | Control | 554 | 0.17 | CC 15; CG 161; GG 378 |  |  |
| ss825678898 | 75,276,401 | Case | 143 | 0.004 | -- 0; -T 1; TT 142 | 0.58 | 0.55 (0.07 - 4.52) |
|  |  | Control | 556 | 0.006 | -- 0; -T 7; TT 549 |  |  |
| rs17010664 | 75,276,290 | Case | 142 | 0.09 | GG 2; AG 20; AA 120 | 0.38 | 0.81 (0.51 - 1.29) |
|  |  | Control | 555 | 0.10 | GG 9; AG 95; AA 451 |  |  |
| rs12713828 | 75,276,081 | Case | 143 | 0.49 | GG 33; AG 73; AA 37 | 0.07 | 1.27 (0.98 - 1.65) |
|  |  | Control | 556 | 0.43 | GG 95; AG 284; AA 177 |  |  |

^1^ SNP, single nucleotide polymorphism. Reference SNP (rs) ID given where available; otherwise Chromosome number_position stated.

^2^ Base position on chromosome 2, hg19 Build NCBI37 May 2009.

^3^ University College London (UCL) bipolar affective disorder (BPAD) 1 subsample of individuals with comorbid alcohol dependence (BPALC). Controls have been screened for a history of mental illness and alcohol use disorders.

^4^ N, number of samples.

^5^ P Value, Probability value determined with Chi-Square analysis unless otherwise specified.

^6^ OR (95 % CI), odds ratio with 95 % confidence intervals in parentheses.

^7^ Data for rs3771829 previously published for BPALC (Lydall et al., 2011).

^8^ rs1106854 is triallelic, Cochrane Armitage Test of Trend (p = 0.274 Pearson Chi-Square 1.782, Linear by linear association 1.194, df = 4, N = 698).

**eTable V: Tests of association with *TACR1* SNPs in the UCL sample of alcohol dependence syndrome relative to screened controls**

| **SNP ID**^1^ | **Position Chr2 (NCBI37/ hg19)**^2^ | **ADS *vs*. Screened Controls**^3^ | **N**^4^ | **Minor Allele Frequency** | **Genotype counts** | ***P* Value**^5^ | **OR (95 % CI)**^6^ |
| --- | --- | --- | --- | --- | --- | --- | --- |
| rs17011370 | 75,692,623 | Case | 965 | 0.05 | TT 3; CT 100; CC 862 | 0.27 | 0.84 (0.62 - 1.15) |
|  |  | Control | 549 | 0.06 | TT 2; CT 67; CC 480 |  |  |
| rs13387833 | 75,428,396 | Case | 966 | 0.08 | TT 6; CT 143; CC 817 | 0.24 | 0.86 (0.66 - 1.11) |
|  |  | Control | 590 | 0.09 | TT 2; CT 105; CC 483 |  |  |
| rs2111375 | 75,427,878 | Case | 970 | 0.29 | AA 82; AG 402; GG 486 | 0.64 | 1.04 (0.88 - 1.22) |
|  |  | Control | 564 | 0.28 | AA 55; AG 210; GG 299 |  |  |
| rs10210648 | 75,426,903 | Case | 980 | 0.08 | CC 7; CG 146; GG 827 | 0.20 | 0.85 (0.66 - 1.09) |
|  |  | Control | 563 | 0.10 | CC 2; CG 103; GG 458 |  |  |
| rs6715729 | 75,425,728 | Case | 977 | 0.47 | GG 224; AG 479; AA 274 | 0.08 | 0.88 (0.76 - 1.02) |
|  |  | Control | 564 | 0.51 | GG 150; AG 272; AA 142 |  |  |
| rs3771856 | 75,414,014 | Case | 970 | 0.45 | GG 202; AG 470; AA 298 | 0.07 | 0.87 (0.75 - 1.01) |
|  |  | Control | 562 | 0.48 | GG 134; AG 277; AA 151 |  |  |
| rs3771833 | 75,366,937 | Case | 975 | 0.11 | TT 12; CT 185; CC 778 | 0.05 | 1.28 (1.00 - 1.65) |
|  |  | Control | 561 | 0.09 | TT 6; CT 84; CC 471 |  |  |
| rs3771829 | 75,364,145 | Case | 966 | 0.09 | GG 10; CG 158; CC 798 | 0.0022 | 1.56 (1.17 - 2.09) |
|  |  | Control | 566 | 0.06 | GG 2; CG 65; CC 499 |  |  |

^1^ SNP, single nucleotide polymorphism. Reference SNP (rs) ID given where available; otherwise Chromosome number_position stated.

^2^ Base position on chromosome 2, hg19 Build NCBI37 May 2009.

^3^ University College London (UCL) alcohol dependence syndrome (ADS) sample. Controls have been screened for a history of mental illness and alcohol use disorders.

^4^ N, number of samples.

^5^ P Value, Probability value determined with Chi-Square analysis unless otherwise specified.

^6^ OR (95 % CI), odds ratio with 95 % confidence intervals in parentheses.

**eTable VI: Tests of association with *TACR1* SNPs in the UCL screened and unscreened controls**

| **SNP ID ^1^** | **Position Chr 2 (NCBI37/hg19)^2^** | **Unscreened *vs*. Screened Controls^3^** | **N**^4^ | **Minor Allele Frequency** | **Genotype counts** | ***P* Value**^5^ | **OR (95 % CI)**^6^ |
| --- | --- | --- | --- | --- | --- | --- | --- |
| rs17011370 | 75,692,623 | Unscreened Control | 380 | 0.06 | TT 0; CT 48; CC 332 | 0.90 | 0.98 (0.67 - 1.42) |
|  |  | Screened Control | 549 | 0.06 | TT 2; CT 67; CC 480 |  |  |
| rs59099335 | 75,429,888 | Unscreened Control | 381 | 0.07 | TT 3; CT 45; CC 333 | 0.26 | 0.81 (0.57 - 1.16) |
|  |  | Screened Control | 549 | 0.08 | TT 6; CT 77; CC 466 |  |  |
| rs34374747 | 75,429,380 | Unscreened Control | 362 | 0.28 | GG 35; AG 135; AA 192 | 0.21 | 1.14 (0.93 - 1.42) |
|  |  | Screened Control | 532 | 0.26 | GG 47; AG 179; AA 306 |  |  |
| rs1477157 | 75,429,228 | Unscreened Control | 379 | 0.48 | AA 97; AG 198; GG 84 | 0.38 | 0.92 (0.77 - 1.11) |
|  |  | Screened Control | 557 | 0.50 | AA 142; AG 269; GG 146 |  |  |
| rs1477156 | 75,429,141 | Unscreened Control | 376 | 0.49 | TT 94; CT 197; CC 85 | 0.27 | 0.90 (0.75 - 1.08) |
|  |  | Screened Control | 559 | 0.51 | TT 134; CT 275; CC 150 |  |  |
| rs13387833 | 75,428,396 | Unscreened Control | 471 | 0.07 | TT 0; CT 66; CC 405 | 0.06 | 0.74 (0.54 - 1.02) |
|  |  | Screened Control | 590 | 0.09 | TT 2; CT 105; CC 483 |  |  |
| rs2111375 | 75,427,878 | Unscreened Control | 379 | 0.30 | AA 33; AG 163; GG 183 | 0.39 | 1.09 (0.89 - 1.34) |
|  |  | Screened Control | 564 | 0.28 | AA 55; AG 210; GG 299 |  |  |
| rs13384011 | 75,427,244 | Unscreened Control | 379 | 0.06 | AA 0; AG 48; GG 331 | 0.012 | 0.64 (0.45 - 0.91) |
|  |  | Screened Control | 559 | 0.10 | AA 2; AG 103; GG 454 |  |  |
| rs2193405 | 75,426,968 | Unscreened Control | 374 | 0.30 | AA 35; AT 154; TT 185 | 0.25 | 1.13 (0.92 - 1.38) |
|  |  | Screened Control | 560 | 0.28 | AA 51; AT 206; TT 303 |  |  |
| rs10210648 | 75,426,903 | Unscreened Control | 380 | 0.06 | CC 0; CG 49; GG 331 | 0.018 | 0.66 (0.46 - 0.93) |
|  |  | Screened Control | 563 | 0.10 | CC 2; CG 103; GG 458 |  |  |
| rs200655774 | 75,426,103 | Unscreened Control | 380 | 0.0013 | CC 0; CG 1; GG 379 | 0.80 | 0.73 (0.07 - 8.09) |
|  |  | Screened Control | 557 | 0.0018 | CC 0; CG 2; GG 555 |  |  |
| rs6715729 | 75,425,728 | Unscreened Control | 377 | 0.49 | GG 83; AG 200; AA 94 | 0.36 | 0.92 (0.76 - 1.10) |
|  |  | Screened Control | 564 | 0.51 | GG 150; AG 272; AA 142 |  |  |
| rs2024512 | 75,425,623 | Unscreened Control | 376 | 0.47 | AA 79; AG 197; GG 100 | 0.49 | 0.94 (0.78 - 1.13) |
|  |  | Screened Control | 559 | 0.49 | AA 137; AG 272; GG 150 |  |  |
| **SNP ID ^1^** | **Position Chr 2 (NCBI37/hg19)^2^** | **Unscreened *vs*. Screened Controls^3^** | **N**^4^ | **Minor Allele Frequency** | **Genotype counts** | ***P* Value**^5^ | **OR (95 % CI)**^6^ |
| rs3771856 | 75,414,014 | Unscreened Control | 375 | 0.49 | GG 87; AG 193; AA 95 | 0.85 | 1.02 (0.85 - 1.23) |
|  |  | Screened Control | 562 | 0.48 | GG 134; AG 277; AA 151 |  |  |
| rs3771833 | 75,366,937 | Unscreened Control | 380 | 0.11 | TT 4; CT 75; CC 301 | 0.09 | 1.31 (0.96 - 1.79) |
|  |  | Screened Control | 561 | 0.09 | TT 6; CT 84; CC 471 |  |  |
| rs3771829 | 75,364,145 | Unscreened Control | 373 | 0.10 | GG 2; CG 69; CC 302 | 0.003 | 1.67 (1.19 - 2.36) |
|  |  | Screened Control | 566 | 0.06 | GG 2; CG 65; CC 499 |  |  |
| rs78052302 | 75,280,721 | Unscreened Control | 380 | 0.05 | -- 1; -T 33; TT 346 | 0.67 | 1.10 (0.70 - 1.72) |
|  |  | Screened Control | 560 | 0.04 | -- 3; -T 41; TT 516 |  |  |
| rs201914096 | 75,278,195 | Unscreened Control | 378 | 0.0013 | TT 0; CT 1; CC 377 | 0.79 | 1.46 (0.09 - 23.39) |
|  |  | Screened Control | 552 | 0.0009 | TT 0; CT 1; CC 551 |  |  |
| rs1106854 | 75,278,014 | Unscreened Control | 381 | G 0.02, T 0.27 | GG 1; TG 2; CG 10; TT 29; CT 143; CC 196 | 0.05^7^ | − |
|  |  | Screened Control | 555 | G 0.03, T 0.29 | GG 0; TG 4; CG 27; TT 49; CT 222; CC 253 |  |  |
| rs881 | 75,276,429 | Unscreened Control | 379 | 0.18 | CC 10; CG 114; GG 255 | 0.81 | 1.03 (0.81 - 1.32) |
|  |  | Screened Control | 554 | 0.17 | CC 15; CG 161; GG 378 |  |  |
| ss825678898 | 75,276,401 | Unscreened Control | 378 | 0.007 | TT 0; -T 5; -- 373 | 0.93 | 1.05 (0.33 - 3.32) |
|  |  | Screened Control | 556 | 0.006 | TT 0; -T 7; -- 549 |  |  |
| rs17010664 | 75,276,290 | Unscreened Control | 376 | 0.11 | AA 4; AG 77; GG 295 | 0.44 | 1.12 (0.83 - 1.52) |
|  |  | Screened Control | 555 | 0.10 | AA 9; AG 95; GG 451 |  |  |
| rs12713828 | 75,276,081 | Unscreened Control | 377 | 0.44 | GG 68; AG 196; AA 113 | 0.55 | 1.06 (0.88 - 1.28) |
|  |  | Screened Control | 556 | 0.43 | GG 95; AG 284; AA 177 |  |  |

^1^ SNP, single nucleotide polymorphism. Reference SNP (rs) ID given where available; otherwise Chromosome number_position stated.

^2^ Base position on chromosome 2, hg19 Build NCBI37 May 2009.

^3^ University College London (UCL) screened controls who have been screened for a history of mental illness and alcohol use disorders and unscreened normal controls.

^4^ N, number of samples.

^5^ P Value, Probability value determined with Chi-Square analysis unless otherwise specified.

^6^ OR (95 % CI), odds ratio with 95 % confidence intervals in parentheses.

^7^ rs1106854 is triallelic, Cochrane Armitage Test of Trend (Pearson Chi-Square 6.830, Linear by linear association 3.913, df = 5, N = 936).

**eTable VII. Significant tests of association with bipolar affective disorder in the UCL1 and UCL2 samples using imputed data from the 1000 genomes project**

| **rs ID**^1^ | **Position Chr2**^2^ | **Imputed SNPs**^3^ | | | | | | |  | | **Variant Effect Predictor**^11^ | | |
| --- | --- | --- | --- | --- | --- | --- | --- | --- | --- | --- | --- | --- | --- |
|  |  | **A1**^4^ | **A2**^5^ | **AMPC**^6^ | **F_A**^7^ | **F_U**^8^ | **P**^9^ | **OR (95 % CI)**^10^ | |  | | **Gene**^12^ | **Consequence of variant**^13^ |
| rs184146027 | 75,213,208 | C | T | 0.98 | 0.03 | 0.05 | 0.007 | 0.57 (0.39 ± 0.84) | |  | | - | Regulatory region: ENSR00001543756; Intergenic variant |
| rs138510182 | 75,218,182 | T | C | 0.98 | 0.03 | 0.05 | 0.01 | 0.58 (0.39 ± 0.86) | |  | | - | Intergenic variant |
| rs7602776 | 75,218,331 | T | A | 1 | 0.48 | 0.44 | 0.04 | 1.2 (1.01 ± 1.44) | |  | | - | Intergenic variant |
| rs7571034 | 75,238,361 | C | G | 1 | 0.48 | 0.43 | 0.04 | 1.21 (1.01 ± 1.45) | |  | | - | Intergenic variant |
| rs13012537 | 75,269,064 | T | C | 1 | 0.47 | 0.42 | 0.02 | 1.23 (1.03 ± 1.46) | |  | | - | Intergenic variant |
| chr2:75280719:D | 75,280,719 | GT | G | 0.98 | 0.002 | 0 | 0.05 | -1 | |  | | *TACR1* | Regulatory region: ENSR00000676664; Intronic: ENST00000409848, ENST00000305249 |
| rs74376589 | 75,281,090 | A | G | 0.98 | 0.002 | 0 | 0.04 | -1 | |  | | *TACR1* | Regulatory region: ENSR00000676664; Intronic: ENST00000409848, ENST00000305249 |
| rs183232680 | 75,297,512 | A | G | 0.97 | 0.08 | 0.05 | 0.007 | 1.52 (1.12 ± 2.07) | |  | | *TACR1* | Intronic: ENST00000409848, ENST00000305249 |
| rs191039988 | 75,306,142 | T | C | 0.99 | 0.001 | 0 | 0.04 | -1 | |  | | *TACR1* | Intronic: ENST00000409848, ENST00000305249 |
| rs79127835 | 75,323,285 | A | G | 0.99 | 0.001 | 0 | 0.04 | -1 | |  | | *TACR1* | Intronic: ENST00000409848, ENST00000305249 |
| rs72918532 | 75,323,874 | G | C | 0.99 | 0.02 | 0.05 | 0.003 | 0.45 (0.28 ± 0.73) | |  | | *TACR1* | Intronic: ENST00000409848, ENST00000305249 |
| rs115249523 | 75,324,553 | A | G | 0.99 | 0.02 | 0.05 | 0.002 | 0.47 (0.3 ± 0.74) | |  | | *TACR1* | Intronic: ENST00000409848, ENST00000305249 |
| rs189985009 | 75,324,929 | A | G | 0.99 | 0.006 | 0.01 | 0.04 | 0.4 (0.18 ± 0.89) | |  | | *TACR1* | Intronic: ENST00000409848, ENST00000305249 |
| rs146950668 | 75,326,286 | C | G | 0.99 | 0.02 | 0.06 | 0.0005 | 0.41 (0.25 ± 0.65) | |  | | *TACR1* | Regulatory region: ENSR00000676666; Intronic: ENST00000409848, ENST00000305249 |
| chr2:75327565:D | 75,327,565 | ACT | A | 0.99 | 0.02 | 0.06 | 0.0002 | 0.38 (0.24 ± 0.61) | |  | | *TACR1* | Regulatory region: ENSR00001543768; Intronic: ENST00000409848, ENST00000305249 |
| rs77446510 | 75,328,309 | A | G | 0.98 | 0.01 | 0.005 | 0.03 | 2.7 (1.05 ± 6.93) | |  | | *TACR1* | Regulatory region: ENSR00001543768; Intronic: ENST00000409848, ENST00000305249 |
| rs182005577 | 75,329,569 | G | C | 0.97 | 0.02 | 0.01 | 0.007 | 2.82 (1.25 ± 6.33) | |  | | *TACR1* | Intronic: ENST00000409848, ENST00000305249 |
| rs187211761 | 75,329,848 | A | G | 0.99 | 0.03 | 0.06 | 0.0006 | 0.43 (0.28 ± 0.67) | |  | | *TACR1* | Intronic: ENST00000409848, ENST00000305249 |
| rs7582109 | 75,334,988 | A | T | 0.99 | 0.0006 | 0.004 | 0.04 | 0.16 (0.02 ± 1.33) | |  | | *TACR1* | Intronic: ENST00000409848, ENST00000305249 |
| rs115963180 | 75,335,317 | T | G | 0.98 | 0.02 | 0.01 | 0.007 | 2.84 (1.27 ± 6.38) | |  | | *TACR1* | Intronic: ENST00000409848, ENST00000305249 |
| rs73935535 | 75,337,524 | A | T | 0.99 | 0.0006 | 0.004 | 0.04 | 0.16 (0.02 ± 1.34) | |  | | *TACR1* | Intronic: ENST00000409848, ENST00000305249 |
| rs189288944 | 75,338,289 | T | C | 0.98 | 0.02 | 0.005 | 0.01 | 3.01 (1.19 ± 7.6) | |  | | *TACR1* | Intronic: ENST00000409848, ENST00000305249 |
| rs141271615 | 75,338,394 | G | T | 0.94 | 0.11 | 0.18 | 0.04 | 0.57 (0.36 ± 0.9) | |  | | *TACR1* | Intronic: ENST00000409848, ENST00000305249 |
| rs72918549 | 75,338,474 | G | A | 0.94 | 0.11 | 0.17 | 0.04 | 0.58 (0.36 ± 0.91) | |  | | *TACR1* | Intronic: ENST00000409848, ENST00000305249 |
| rs139788317 | 75,338,631 | A | T | 0.99 | 0.03 | 0.06 | 0.0002 | 0.4 (0.26 ± 0.62) | |  | | *TACR1* | Intronic: ENST00000409848, ENST00000305249 |
| rs140181726 | 75,339,051 | C | G | 0.98 | 0.02 | 0.006 | 0.03 | 2.55 (1.06 ± 6.12) | |  | | *TACR1* | Intronic: ENST00000409848, ENST00000305249 |
| chr2:75339547:D | 75,339,547 | TG | T | 0.98 | 0.02 | 0.006 | 0.02 | 2.72 (1.14 ± 6.5) | |  | | *TACR1* | Intronic: ENST00000409848, ENST00000305249 |
| rs114427038 | 75,340,504 | T | C | 0.99 | 0.04 | 0.07 | 0.01 | 0.57 (0.4 ± 0.83) | |  | | *TACR1* | Intronic: ENST00000409848, ENST00000305249 |
| rs181219209 | 75,340,807 | G | A | 0.99 | 0.04 | 0.07 | 0.02 | 0.59 (0.41 ± 0.86) | |  | | *TACR1* | Intronic: ENST00000409848, ENST00000305249 |
| rs73935540 | 75,342,181 | A | C | 1 | 0.001 | 0 | 0.04 | -1 | |  | | *TACR1* | Downstream: ENST00000497764; Intronic: ENST00000409848, ENST00000305249 |
| rs184935933 | 75,345,595 | A | T | 0.99 | 0.05 | 0.07 | 0.05 | 0.65 (0.46 ± 0.93) | |  | | *TACR1* | Downstream: ENST00000497764; Intronic: ENST00000409848, ENST00000305249 |
| rs72918570 | 75,348,139 | C | G | 0.99 | 0.05 | 0.07 | 0.05 | 0.65 (0.46 ± 0.93) | |  | | *TACR1* | Upstream: ENST00000497764; Intronic: ENST00000409848, ENST00000305249 |
| rs76517971 | 75,348,161 | T | C | 0.99 | 0.05 | 0.07 | 0.05 | 0.65 (0.46 ± 0.93) | |  | | *TACR1* | Upstream: ENST00000497764; Intronic: ENST00000409848, ENST00000305249 |
| rs7588326 | 75,349,046 | C | T | 0.99 | 0.05 | 0.07 | 0.05 | 0.65 (0.46 ± 0.93) | |  | | *TACR1* | Upstream: ENST00000497764; Intronic: ENST00000409848, ENST00000305249 |
| rs191875370 | 75,349,682 | A | G | 0.98 | 0.01 | 0.006 | 0.03 | 2.62 (1.02 ± 6.71) | |  | | *TACR1* | Upstream: ENST00000497764; Intronic: ENST00000409848, ENST00000305249 |
| rs193032314 | 75,351,125 | C | T | 0.98 | 0.007 | 0.002 | 0.02 | 4.92 (1.08 ± 22.52) | |  | | *TACR1* | Upstream: ENST00000497764; Intronic: ENST00000409848, ENST00000305249 |
| rs147834749 | 75,354,004 | G | A | 0.99 | 0.0006 | 0.004 | 0.04 | 0.16 (0.02 ± 1.32) | |  | | *TACR1* | Intronic: ENST00000409848, ENST00000305249 |
| rs76775346 | 75,355,554 | G | A | 0.96 | 0.01 | 0.004 | 0.04 | 3.43 (0.94 ± 12.52) | |  | | *TACR1* | Intronic: ENST00000409848, ENST00000305249 |
| **rs ID**^1^ | **Position Chr2**^2^ | **Imputed SNPs**^3^ | | | | | | | |  | | **Variant Effect Predictor**^11^ | |
|  |  | **A1**^4^ | **A2**^5^ | **AMPC**^6^ | **F_A**^7^ | **F_U**^8^ | **P**^9^ | **OR (95 % CI)**^10^ | |  | | **Gene**^12^ | **Consequence of variant**^13^ |
| rs185595660 | 75,356,474 | T | G | 0.96 | 0.01 | 0.004 | 0.04 | 3.42 (0.94 ± 12.5) | |  | | *TACR1* | Intronic: ENST00000409848, ENST00000305249 |
| rs181192058 | 75,357,873 | C | T | 0.98 | 0.008 | 0.002 | 0.02 | 4.89 (1.07 ± 22.38) | |  | | *TACR1* | Intronic: ENST00000409848, ENST00000305249 |
| chr2:75359623:D | 75,359,623 | ACTC | A | 0.99 | 0.05 | 0.07 | 0.05 | 0.65 (0.46 ± 0.93) | |  | | *TACR1* | Intronic: ENST00000409848, ENST00000305249 |
| rs114063455 | 75,361,691 | T | C | 0.99 | 0.05 | 0.07 | 0.05 | 0.65 (0.46 ± 0.93) | |  | | *TACR1* | Intronic: ENST00000409848, ENST00000305249 |
| rs3771827 | 75,361,864 | T | C | 0.98 | 0.008 | 0.002 | 0.05 | 3.28 (0.9 ± 11.94) | |  | | *TACR1* | Intronic: ENST00000409848, ENST00000305249 |
| rs741420 | 75,363,874 | T | C | 0.96 | 0.03 | 0.01 | 0.04 | 1.99 (1.01 ± 3.91) | |  | | *TACR1* | Intronic: ENST00000409848, ENST00000305249 |
| rs143757550 | 75,364,259 | G | T | 0.98 | 0.007 | 0.002 | 0.05 | 3.28 (0.9 ± 11.94) | |  | | *TACR1* | Intronic: ENST00000409848, ENST00000305249 |
| rs7581675 | 75,364,685 | A | G | 1 | 0.001 | 0 | 0.04 | -1 | |  | | *TACR1* | Intronic: ENST00000409848, ENST00000305249 |
| rs144018010 | 75,365,301 | A | G | 0.97 | 0.008 | 0.003 | 0.02 | 3.26 (1.06 ± 10.01) | |  | | *TACR1* | Intronic: ENST00000409848, ENST00000305249 |
| rs192905448 | 75,366,948 | G | A | 0.98 | 0.007 | 0.02 | 0.03 | 0.4 (0.18 ± 0.89) | |  | | *TACR1* | Intronic: ENST00000409848, ENST00000305249 |
| rs185152963 | 75,367,534 | A | G | 0.97 | 0.009 | 0.003 | 0.04 | 2.76 (0.99 ± 7.69) | |  | | *TACR1* | Intronic: ENST00000409848, ENST00000305249 |
| rs58913381 | 75,373,021 | A | G | 0.98 | 0.008 | 0.02 | 0.05 | 0.43 (0.2 ± 0.94) | |  | | *TACR1* | Intronic: ENST00000409848, ENST00000305249 |
| rs188368822 | 75,373,164 | C | T | 1 | 0.0009 | 0.004 | 0.03 | 0.22 (0.05 ± 1.02) | |  | | *TACR1* | Intronic: ENST00000409848, ENST00000305249 |
| rs113702328 | 75,377,852 | A | G | 0.97 | 0.01 | 0.004 | 0.03 | 3.13 (1.02 ± 9.64) | |  | | *TACR1* | Intronic: ENST00000409848, ENST00000305249 |
| rs182967369 | 75,383,347 | A | G | 0.96 | 0.08 | 0.04 | 0.04 | 2.09 (1.09 ± 3.99) | |  | | *TACR1* | Intronic: ENST00000409848, ENST00000305249 |
| rs147031506 | 75,384,575 | A | G | 0.98 | 0.1 | 0.14 | 0.01 | 0.64 (0.45 ± 0.89) | |  | | *TACR1* | Regulatory region: ENSR00000593127; Intronic: ENST00000409848, ENST00000305249 |
| rs181659924 | 75,387,720 | C | T | 0.98 | 0.1 | 0.14 | 0.03 | 0.67 (0.48 ± 0.94) | |  | | *TACR1* | Intronic: ENST00000409848, ENST00000305249 |
| rs145228980 | 75,388,944 | C | G | 0.98 | 0.09 | 0.13 | 0.04 | 0.68 (0.48 ± 0.96) | |  | | *TACR1* | Intronic: ENST00000409848, ENST00000305249 |
| rs147054223 | 75,390,022 | A | G | 0.97 | 0.09 | 0.15 | 0.02 | 0.6 (0.41 ± 0.88) | |  | | *TACR1* | Intronic: ENST00000409848, ENST00000305249 |
| rs2422148 | 75,395,997 | C | G | 0.96 | 0.05 | 0.09 | 0.04 | 0.56 (0.34 ± 0.94) | |  | | *TACR1* | Intronic: ENST00000409848, ENST00000305249 |
| rs139759288 | 75,398,465 | C | A | 0.96 | 0.02 | 0.03 | 0.05 | 0.53 (0.29 ± 0.98) | |  | | *TACR1* | Intronic: ENST00000409848, ENST00000305249 |
| rs12469836 | 75,402,895 | C | T | 0.97 | 0.009 | 0.02 | 0.02 | 0.46 (0.24 ± 0.9) | |  | | *TACR1* | Intronic: ENST00000409848, ENST00000305249 |
| rs187148246 | 75,404,008 | C | T | 0.98 | 0.02 | 0.01 | 0.02 | 0.41 (0.18 ± 0.9) | |  | | *TACR1* | Intronic: ENST00000409848, ENST00000305249 |
| rs72920676 | 75,408,094 | C | T | 0.96 | 0 | 0.004 | 0.009 | -1 | |  | | *TACR1* | Intronic: ENST00000409848, ENST00000305249 |
| rs151313513 | 75,408,212 | A | C | 0.95 | 0.02 | 0.05 | 0.04 | 0.44 (0.19 ± 1) | |  | | *TACR1* | Intronic: ENST00000409848, ENST00000305249 |
| rs186366225 | 75,415,434 | T | C | 0.95 | 0.18 | 0.09 | 0.002 | 2.29 (1.39 ± 3.76) | |  | | *TACR1* | Intronic: ENST00000409848, ENST00000305249 |
| rs190748962 | 75,415,485 | C | T | 0.95 | 0.17 | 0.09 | 0.002 | 2.27 (1.39 ± 3.72) | |  | | *TACR1* | Intronic: ENST00000409848, ENST00000305249 |
| rs141945058 | 75,423,409 | G | A | 0.99 | 0.03 | 0.05 | 0.02 | 0.63 (0.42 ± 0.94) | |  | | *TACR1* | Intronic: ENST00000409848, ENST00000305249 |
| rs186012981 | 75,424,320 | A | C | 0.98 | 0.006 | 0.001 | 0.006 | 5.86 (1.3 ± 26.48) | |  | | *TACR1* | Intronic: ENST00000409848, ENST00000305249 |
| rs112389239 | 75,427,459 | A | G | 0.99 | 0.03 | 0.05 | 0.03 | 0.66 (0.45 ± 0.98) | |  | | *TACR1* | Regulatory region: ENSR00001543775; Upstream: ENST00000409848, ENST00000305249 |
| rs114752002 | 75,428,596 | T | C | 0.99 | 0.03 | 0.04 | 0.04 | 0.66 (0.43 ± 0.99) | |  | | *TACR1* | Upstream: ENST00000409848, ENST00000305249 |
| rs6731496 | 75,429,304 | A | G | 0.99 | 0.03 | 0.04 | 0.03 | 0.64 (0.42 ± 0.97) | |  | | *TACR1* | Upstream: ENST00000409848, ENST00000305249 |
| rs114884290 | 75,431,258 | A | G | 0.99 | 0.02 | 0.04 | 0.03 | 0.61 (0.39 ± 0.96) | |  | | *TACR1* | Upstream: ENST00000305249 |
| rs11678703 | 75,435,188 | C | A | 0.99 | 0.02 | 0.03 | 0.03 | 0.59 (0.36 ± 0.96) | |  | | - | Intergenic variant |
| rs137974296 | 75,435,288 | G | A | 0.99 | 0.01 | 0.03 | 0.02 | 0.54 (0.32 ± 0.93) | |  | | - | Intergenic variant |
| rs147418190 | 75,436,595 | G | T | 0.99 | 0.02 | 0.03 | 0.01 | 0.55 (0.33 ± 0.9) | |  | | - | Intergenic variant |
| rs145257418 | 75,438,645 | C | G | 0.98 | 0.01 | 0.02 | 0.03 | 0.55 (0.31 ± 0.97) | |  | | - | Intergenic variant |
| rs114566962 | 75,439,295 | T | C | 0.98 | 0.02 | 0.03 | 0.006 | 0.49 (0.29 ± 0.83) | |  | | - | Intergenic variant |
| rs115538723 | 75,439,580 | A | T | 0.97 | 0.06 | 0.09 | 0.05 | 0.68 (0.47 ± 0.98) | |  | | - | Intergenic variant |
| rs188610938 | 75,440,372 | C | T | 0.99 | 0.003 | 0.009 | 0.05 | 0.37 (0.13 ± 1.05) | |  | | - | Intergenic variant |
| rs75677014 | 75,440,438 | G | A | 0.99 | 0.003 | 0.009 | 0.02 | 0.3 (0.1 ± 0.92) | |  | | - | Intergenic variant |
| rs79656330 | 75,440,536 | A | G | 0.99 | 0.003 | 0.009 | 0.01 | 0.28 (0.09 ± 0.85) | |  | | - | Intergenic variant |
| **rs ID**^1^ | **Position Chr2**^2^ | **Imputed SNPs**^3^ | | | | | | | |  | | **Variant Effect Predictor**^11^ | |
|  |  | **A1**^4^ | **A2**^5^ | **AMPC**^6^ | **F_A**^7^ | **F_U**^8^ | **P**^9^ | **OR (95 % CI)**^10^ | |  | | **Gene**^12^ | **Consequence of variant**^13^ |
| rs141051573 | 75,440,568 | T | C | 0.99 | 0.003 | 0.009 | 0.01 | 0.28 (0.09 ± 0.85) | |  | | - | Intergenic variant |
| rs181569822 | 75,442,305 | C | T | 0.97 | 0.04 | 0.06 | 0.04 | 0.65 (0.43 ± 0.98) | |  | | - | Intergenic variant |
| rs186625011 | 75,442,359 | C | T | 0.97 | 0.07 | 0.1 | 0.04 | 0.71 (0.51 ± 0.99) | |  | | - | Intergenic variant |
| rs13009811 | 75,442,851 | G | A | 0.98 | 0.02 | 0.05 | 0.007 | 0.52 (0.32 ± 0.85) | |  | | - | Intergenic variant |
| rs190194915 | 75,442,949 | G | A | 0.97 | 0.008 | 0.03 | 0.0002 | 0.28 (0.13 ± 0.58) | |  | | - | Intergenic variant |
| rs186770770 | 75,442,994 | G | T | 0.98 | 0.015 | 0.03 | 0.006 | 0.43 (0.23 ± 0.79) | |  | | - | Intergenic variant |
| rs7423476 | 75,443,305 | G | T | 0.98 | 0.01 | 0.02 | 0.04 | 0.53 (0.28 ± 0.98) | |  | | - | Intergenic variant |
| rs13016171 | 75,443,569 | C | T | 0.98 | 0.004 | 0.0006 | 0.02 | 7.46 (0.92 ± 60.74) | |  | | - | Intergenic variant |
| rs193175250 | 75,443,585 | G | A | 0.97 | 0.06 | 0.09 | 0.05 | 0.66 (0.44 ± 1) | |  | | - | Intergenic variant |
| rs185031520 | 75,443,633 | G | C | 0.97 | 0.03 | 0.06 | 0.02 | 0.5 (0.28 ± 0.89) | |  | | - | Intergenic variant |
| rs112638930 | 75,443,694 | T | A | 0.97 | 0.04 | 0.08 | 0.0007 | 0.44 (0.26 ± 0.72) | |  | | - | Intergenic variant |
| rs11684394 | 75,444,073 | C | T | 0.97 | 0.04 | 0.07 | 0.006 | 0.52 (0.32 ± 0.84) | |  | | - | Intergenic variant |
| rs185761087 | 75,461,190 | G | T | 0.97 | 0.002 | 0.007 | 0.02 | 0.21 (0.04 ± 0.97) | |  | | - | Intergenic variant |
| rs189422191 | 75,467,598 | T | C | 0.95 | 0.05 | 0.14 | 0.02 | 0.31 (0.12 ± 0.81) | |  | | - | Intergenic variant |
| rs7576919 | 75,471,057 | C | T | 0.95 | 0.007 | 0.03 | 0.03 | 0.22 (0.05 ± 1.06) | |  | | - | Intergenic variant |
| rs11679945 | 75,478,876 | A | G | 0.95 | 0.006 | 0.03 | 0.04 | 0.22 (0.05 ± 1.07) | |  | | - | Intergenic variant |
| rs190565466 | 75,489,671 | A | T | 0.95 | 0.005 | 0.02 | 0.02 | 0.21 (0.04 ± 0.98) | |  | | - | Intergenic variant |
| rs148649131 | 75,493,414 | G | A | 1 | 0 | 0.002 | 0.02 | -1 | |  | | - | Intergenic variant |
| rs181686281 | 75,498,735 | C | T | 1 | 0 | 0.002 | 0.02 | -1 | |  | | - | Intergenic variant |
| rs184324836 | 75,502,108 | G | A | 0.99 | 0 | 0.003 | 0.009 | -1 | |  | | - | Intergenic variant |
| rs60026310 | 75,505,129 | A | C | 1 | 0.001 | 0 | 0.04 | -1 | |  | | - | Intergenic variant |
| rs183565564 | 75,508,345 | A | T | 0.98 | 0.12 | 0.1 | 0.04 | 1.32 (1.01 ± 1.73) | |  | | - | Intergenic variant |
| rs17581300 | 75,517,499 | C | G | 1 | 0.14 | 0.17 | 0.02 | 0.75 (0.59 ± 0.96) | |  | | - | Intergenic variant |
| rs181904155 | 75,520,999 | A | C | 1 | 0 | 0.002 | 0.008 | -1 | |  | | - | Intergenic variant |
| rs13405891 | 75,521,579 | A | T | 0.98 | 0.002 | 0 | 0.04 | -1 | |  | | - | Regulatory region: ENSR00001543779; Intergenic variant |
| rs75634886 | 75,521,877 | A | G | 0.99 | 0.002 | 0 | 0.008 | -1 | |  | | - | Intergenic variant |
| rs114705479 | 75,522,809 | A | C | 0.99 | 0.002 | 0 | 0.008 | -1 | |  | | - | Intergenic variant |
| rs183586034 | 75,522,938 | G | A | 1 | 0 | 0.002 | 0.008 | -1 | |  | | - | Intergenic variant |
| rs72924325 | 75,529,966 | T | G | 1 | 0.001 | 0 | 0.04 | -1 | |  | | - | Intergenic variant |
| rs146267303 | 75,533,387 | G | A | 1 | 0 | 0.002 | 0.008 | -1 | |  | | - | Intergenic variant |
| rs76075549 | 75,534,966 | C | G | 0.99 | 0.002 | 0 | 0.008 | -1 | |  | | - | Intergenic variant |
| rs151109869 | 75,544,006 | C | T | 0.99 | 0.002 | 0 | 0.008 | -1 | |  | | - | Intergenic variant |
| rs142857411 | 75,545,510 | G | T | 0.99 | 0.002 | 0 | 0.008 | -1 | |  | | - | Intergenic variant |
| rs189005029 | 75,546,367 | C | T | 1 | 0 | 0.002 | 0.008 | -1 | |  | | - | Intergenic variant |
| rs185335542 | 75,548,794 | G | T | 0.99 | 0.003 | 0 | 0.008 | -1 | |  | | - | Intergenic variant |
| rs189006069 | 75,550,354 | A | G | 1 | 0.001 | 0 | 0.04 | -1 | |  | | - | Intergenic variant |
| rs11904081 | 75,551,477 | G | A | 1 | 0 | 0.002 | 0.008 | -1 | |  | | - | Regulatory region: ENSR00001543783; Intergenic variant |
| rs141833018 | 75,556,648 | A | G | 0.99 | 0.003 | 0 | 0.004 | -1 | |  | | - | Intergenic variant |
| rs150709943 | 75,557,609 | C | T | 0.99 | 0.001 | 0 | 0.04 | -1 | |  | | - | Intergenic variant |
| rs147291362 | 75,561,289 | C | T | 0.99 | 0.001 | 0 | 0.04 | -1 | |  | | - | Intergenic variant |
| chr2:75561695:D | 75,561,695 | TG | T | 0.99 | 0.003 | 0 | 0.004 | -1 | |  | | - | Intergenic variant |
| rs10194748 | 75,564,912 | A | G | 1 | 0.16 | 0.21 | 0.02 | 0.76 (0.6 ± 0.95) | |  | | - | Intergenic variant |
| **rs ID**^1^ | **Position Chr2**^2^ | **Imputed SNPs**^3^ | | | | | | | |  | | **Variant Effect Predictor**^11^ | |
|  |  | **A1**^4^ | **A2**^5^ | **AMPC**^6^ | **F_A**^7^ | **F_U**^8^ | **P**^9^ | **OR (95 % CI)**^10^ | |  | | **Gene**^12^ | **Consequence of variant**^13^ |
| rs117771081 | 75,569,397 | C | T | 0.99 | 0.001 | 0 | 0.04 | -1 | |  | | - | Intergenic variant |
| rs142287196 | 75,571,000 | T | C | 1 | 0.002 | 0 | 0.02 | -1 | |  | | - | Intergenic variant |
| rs13385597 | 75,571,004 | A | G | 1 | 0.14 | 0.18 | 0.01 | 0.73 (0.58 ± 0.93) | |  | | - | Intergenic variant |
| rs191141277 | 75,579,348 | G | T | 0.99 | 0.001 | 0 | 0.04 | -1 | |  | | - | Intergenic variant |
| rs6755981 | 75,583,549 | C | T | 1 | 0.12 | 0.16 | 0.02 | 0.74 (0.57 ± 0.95) | |  | | - | Intergenic variant |
| rs188600432 | 75,585,821 | C | T | 0.99 | 0.001 | 0 | 0.04 | -1 | |  | | - | Intergenic variant |
| rs192252187 | 75,590,706 | C | T | 0.99 | 0.001 | 0 | 0.04 | -1 | |  | | - | Intergenic variant |
| rs181673566 | 75,590,952 | G | C | 0.99 | 0.001 | 0 | 0.04 | -1 | |  | | - | Intergenic variant |
| rs115401533 | 75,594,937 | G | A | 0.99 | 0.002 | 0 | 0.04 | -1 | |  | | - | Intergenic variant |
| rs189569259 | 75,595,918 | A | C | 0.99 | 0.002 | 0 | 0.04 | -1 | |  | | - | Intergenic variant |
| rs183027190 | 75,616,156 | G | A | 0.96 | 0.02 | 0.04 | 0.04 | 0.41 (0.17 ± 1.02) | |  | | - | Intergenic variant |
| rs146167951 | 75,616,203 | A | G | 0.96 | 0.02 | 0.04 | 0.04 | 0.4 (0.16 ± 1) | |  | | - | Intergenic variant |
| rs149834339 | 75,616,229 | G | C | 0.96 | 0.02 | 0.04 | 0.04 | 0.4 (0.16 ± 1) | |  | | - | Intergenic variant |
| rs190379579 | 75,617,484 | G | C | 0.99 | 0.002 | 0 | 0.04 | -1 | |  | | - | Regulatory region: ENSR00001045343; Intergenic variant |
| rs2110935 | 75,617,583 | G | A | 1 | 0.49 | 0.44 | 0.03 | 1.22 (1.02 ± 1.45) | |  | | - | Regulatory region: ENSR00001045343; Intergenic variant |
| rs4465789 | 75,618,460 | T | A | 0.99 | 0.002 | 0 | 0.04 | -1 | |  | | - | Intergenic variant |
| chr2:75618722:I | 75,618,722 | T | TG | 0.99 | 0.002 | 0 | 0.04 | -1 | |  | | - | Intergenic variant |
| rs72908442 | 75,619,409 | A | C | 0.99 | 0.002 | 0 | 0.04 | -1 | |  | | - | Intergenic variant |
| rs13389037 | 75,629,478 | C | T | 0.98 | 0 | 0.003 | 0.02 | -1 | |  | | - | Intergenic variant |
| rs1160613 | 75,632,552 | G | T | 1 | 0.03 | 0.01 | 0.009 | 2.3 (1.19 ± 4.45) | |  | | - | Intergenic variant |
| rs7567151 | 75,633,124 | C | A | 1 | 0.03 | 0.01 | 0.01 | 2.28 (1.18 ± 4.41) | |  | | - | Intergenic variant |
| rs187459035 | 75,636,503 | G | A | 0.96 | 0 | 0.003 | 0.02 | -1 | |  | | - | Intergenic variant |
| rs78376082 | 75,638,049 | T | C | 0.96 | 0 | 0.003 | 0.02 | -1 | |  | | - | Intergenic variant |
| rs1159872 | 75,638,918 | C | T | 0.96 | 0 | 0.002 | 0.02 | -1 | |  | | - | Intergenic variant |
| rs144567764 | 75,665,578 | C | T | 0.98 | 0.0006 | 0.004 | 0.05 | 6 (0.72 ± 49.93) | |  | | - | Intergenic variant |
| rs192870474 | 75,672,080 | T | G | 0.95 | 0.03 | 0.08 | 0.03 | 2.76 (1.26 ± 6.05) | |  | | - | Intergenic variant |
| rs80160138 | 75,677,315 | A | G | 0.95 | 0.006 | 0.04 | 0.007 | 6.19 (1.37 ± 27.89) | |  | | - | Intergenic variant |
| rs72814734 | 75,683,308 | T | C | 0.97 | 0.13 | 0.19 | 0.03 | 0.64 (0.44 ± 0.92) | |  | | *GAPDHP57* | Non coding exon variant in ENST00000424400, position 397 in cDNA |
| rs77637660 | 75,688,679 | T | C | 0.97 | 0.13 | 0.19 | 0.01 | 0.62 (0.43 ± 0.88) | |  | | *GAPDHP57* | Upstream of ENST00000424400 |
| rs148204204 | 75,689,855 | G | C | 0.98 | 0.09 | 0.15 | 0.004 | 0.61 (0.44 ± 0.84) | |  | | - | Intergenic variant |
| rs188247076 | 75,691,402 | G | T | 0.98 | 0.1 | 0.15 | 0.004 | 0.62 (0.45 ± 0.85) | |  | | - | Intergenic variant |
| rs191379486 | 75,692,291 | G | A | 0.98 | 0.07 | 0.1 | 0.007 | 0.65 (0.48 ± 0.87) | |  | | *FAM176A* | Downstream of ENST00000485891, ENST00000490746 |
| chr2:75693696:D | 75,693,696 | AC | A | 0.98 | 0.07 | 0.1 | 0.01 | 0.67 (0.49 ± 0.9) | |  | | *FAM176A* | Downstream of ENST00000485891, ENST00000490746 |
| rs150650642 | 75,694,706 | C | T | 0.98 | 0.07 | 0.1 | 0.01 | 0.67 (0.5 ± 0.9) | |  | | *FAM176A* | Downstream of ENST00000485891, ENST00000490746 |
| rs6756306 | 75,699,096 | G | A | 0.99 | 0.004 | 0.0006 | 0.03 | 6.96 (0.85 ± 56.6) | |  | | *FAM176A* | Upstream of ENST00000444852; Intronic: ENST00000485891, ENST00000490746 |
| rs6756748 | 75,699,657 | C | T | 0.98 | 0.1 | 0.15 | 0.005 | 0.63 (0.46 ± 0.86) | |  | | *FAM176A* | Upstream of ENST00000444852; Intronic: ENST00000485891, ENST00000490746 |
| rs190745719 | 75,699,981 | C | T | 0.97 | 0.07 | 0.1 | 0.005 | 0.63 (0.46 ± 0.86) | |  | | *FAM176A* | Upstream of ENST00000444852; Intronic: ENST00000485891, ENST00000490746 |

^1^ Reference SNP, single nucleotide polymorphism, (rs) ID given; I, insertion, D, deletion.

^2^ Position on Chromosome 2, hg19 Build NCBI37, May 2009.

^3^ Imputed SNPs, using IMPUTE2 software to generate tests of association (Howie et al., 2009; Howie et al., 2011).

^4^ A1, First Allele.

^5^ A2, Second Allele.

^6^ AMPC, average maximum posterior call, which is the probability across all individuals in the sample that are used for the test at each SNP. This is a measure of how much uncertainty there is at each SNP. Samples excluded will be (a) those excluded using the -exclude_samples option, (b) samples with a missing phenotype or covariate relevant to the test, (c) samples without genotypes if the -method threshold option is used, (d) samples where the sum of the genotype probabilities is less than 0.1 (Marchini and Howie 2010).

^7^ F_A, Allele frequency in the affected individuals

^8^ F_U, Allele frequency in the unaffected individuals

^9^ P value, Significance value of case/control association test using the phenotype frequentist additive model P value (≤ 0.05) tested at each SNP versus a model of no association. The beta estimates of the additive model increase in log-odds that can be attributed to each copy of allele 2. When a model cannot be fitted to the data the p-value is set to -1 (Marchini and Howie 2010).

^10^ OR (95 % CI), odds ratio with 95 % confidence intervals in parentheses; -1: metric was not calculated by the IMPUTE2 software.

^11^ Variant Effect Predictor, server used to identify the functional effects of each significantly associated imputed SNP (McLaren 2010).

^12^ Gene, *TACR1* Ensembl ID ENSG00000115353; *GAPDHP57* Ensembl ID ENSG00000236167, a non-codig processed pseudogene; *FAM176A* Ensembl ID ENSG00000115363, *Homo sapiens* family with sequence similarity 176, member A.

^13^ Consequence of variant, notes about where and in which Ensembl transcript (ENST) or Ensembl regulatory region (ENSR) each imputed SNP variant is located. All intergenic and intronic regulatory region variants are located in regions which show enrichment of the H3K27Ac histone mark, which is the acetylation of lysine 27 of the H3 histone protein, according to the ENCODE regulation supertrack on UCSC genome browser (ENCODE Project Consortium 2011).

**eTable VIII. Significant tests of association in comorbid bipolar affective disorder and alcohol dependence in the UCL1 subsample using imputed data from the 1000 genomes project**

| **rs ID**^1^ | **Position Chr2**^2^ | **Imputed SNPs**^3^ | | | | | | |  | **Variant Effect Predictor**^11^ | |
| --- | --- | --- | --- | --- | --- | --- | --- | --- | --- | --- | --- |
|  |  | **A1**^4^ | **A2**^5^ | **AMPC**^6^ | **F_A**^7^ | **F_U**^8^ | **P**^9^ | **OR (95 % CI)**^10^ |  | **Gene**^12^ | **Consequence of variant**^13^ |
| rs3886110 | 75,207,832 | G | T | 1.00 | 0.50 | 0.43 | 0.05 | 1.31 (1 ± 1.7) |  | - | Intergenic |
| rs79756761 | 75,228,055 | G | A | 0.99 | 0.02 | 0.002 | 0.02 | 7.17 (1.31 ± 39.36) |  | - | Intergenic |
| rs7590756 | 75,261,165 | A | G | 0.99 | 0.02 | 0.002 | 0.02 | 6.91 (1.26 ± 37.99) |  | - | Intergenic |
| rs74528910 | 75,261,185 | T | G | 0.99 | 0.02 | 0.003 | 0.02 | 6.8 (1.24 ± 37.42) |  | - | Intergenic |
| chr2:75263603:D | 75,263,603 | TA | T | 0.99 | 0.02 | 0.003 | 0.02 | 6.78 (1.23 ± 37.28) |  | - | Intergenic |
| rs11126450 | 75,264,762 | T | A | 0.99 | 0.02 | 0.002 | 0.02 | 7.19 (1.31 ± 39.5) |  | - | Intergenic |
| rs4614953 | 75,268,803 | T | C | 0.99 | 0.02 | 0.003 | 0.02 | 6.91 (1.26 ± 38.01) |  | - | Intergenic |
| rs12713828 | 75,276,081 | G | A | 1.00 | 0.51 | 0.42 | 0.004 | 0.68 (0.52 ± 0.89) |  | *TACR1* | Downstream: ENST00000305249, ENST00000409848 |
| rs34117315 | 75,276,649 | C | T | 0.99 | 0 | 0.02 | 0.007 | -1 |  | *TACR1* | Synonymous: Ex 5 ENST0000030524; Downstream: ENST00000409848 |
| rs6747372 | 75,280,196 | G | T | 0.98 | 0.02 | 0.002 | 0.02 | 7.18 (1.31 ± 39.46) |  | *TACR1* | Intronic: ENST00000305249, ENST00000409848 |
| chr2:75280825 | 75,280,825 | A | G | 0.99 | 0 | 0.01 | 0.05 | -1 |  | *TACR1* | Synonymous: Ex 3 ENST00000305249/409848; Regulatory region: ENSR00000676664 |
| rs192998916 | 75,282,212 | A | G | 0.98 | 0.02 | 0.002 | 0.02 | 7.18 (1.31 ± 39.46) |  | *TACR1* | Regulatory region: ENCODE; Intronic: ENST00000305249, ENST00000409848 |
| rs143577649 | 75,283,950 | A | G | 0.97 | 0.02 | 0.003 | 0.02 | 7.03 (1.28 ± 38.73) |  | *TACR1* | Regulatory region: ENCODE; Intronic: ENST00000305249, ENST00000409848 |
| rs142594567 | 75,284,600 | G | A | 0.98 | 0.02 | 0.002 | 0.02 | 7.14 (1.3 ± 39.22) |  | *TACR1* | Regulatory region: ENCODE; Intronic: ENST00000305249, ENST00000409848 |
| rs10202481 | 75,288,700 | C | T | 0.99 | 0.02 | 0.002 | 0.02 | 7.32 (1.33 ± 40.18) |  | *TACR1* | Intronic: ENST00000305249, ENST00000409848 |
| rs187231317 | 75,290,307 | G | C | 0.97 | 0.03 | 0.004 | 0.02 | 6.97 (1.26 ± 38.46) |  | *TACR1* | Intronic: ENST00000305249, ENST00000409848 |
| rs55958513 | 75,293,858 | C | G | 0.99 | 0.02 | 0.002 | 0.02 | 7.26 (1.32 ± 39.89) |  | *TACR1* | Intronic: ENST00000305249, ENST00000409848 |
| rs116648770 | 75,295,500 | G | A | 0.99 | 0.02 | 0.002 | 0.02 | 7.26 (1.32 ± 39.89) |  | *TACR1* | Intronic: ENST00000305249, ENST00000409848 |
| rs183232680 | 75,297,512 | A | G | 0.97 | 0.12 | 0.07 | 0.05 | 1.75 (1.02 ± 3) |  | *TACR1* | Intronic: ENST00000305249, ENST00000409848 |
| rs111282986 | 75,298,285 | A | T | 0.98 | 0.02 | 0.002 | 0.02 | 7.13 (1.3 ± 39.18) |  | *TACR1* | Intronic: ENST00000305249, ENST00000409848 |
| chr2:75298299:D | 75,298,299 | GGCCTCTTAGA | G | 0.98 | 0.02 | 0.002 | 0.02 | 7.34 (1.34 ± 40.35) |  | *TACR1* | Intronic: ENST00000305249, ENST00000409848 |
| rs6757924 | 75,298,469 | G | A | 0.98 | 0.02 | 0.002 | 0.02 | 7.13 (1.3 ± 39.18) |  | *TACR1* | Intronic: ENST00000305249, ENST00000409848 |
| rs147246247 | 75,298,494 | A | G | 0.98 | 0.02 | 0.002 | 0.02 | 7.13 (1.3 ± 39.18) |  | *TACR1* | Intronic: ENST00000305249, ENST00000409848 |
| rs114376511 | 75,298,604 | C | T | 0.98 | 0.02 | 0.002 | 0.02 | 7.13 (1.3 ± 39.18) |  | *TACR1* | Intronic: ENST00000305249, ENST00000409848 |
| rs114423962 | 75,299,479 | G | A | 0.98 | 0.02 | 0.002 | 0.02 | 7.31 (1.33 ± 40.16) |  | *TACR1* | Intronic: ENST00000305249, ENST00000409848 |
| rs140908481 | 75,300,999 | C | T | 0.98 | 0.02 | 0.002 | 0.02 | 7.16 (1.3 ± 39.33) |  | *TACR1* | Intronic: ENST00000305249, ENST00000409848 |
| rs191226317 | 75,301,903 | G | A | 0.98 | 0.02 | 0.002 | 0.02 | 7.22 (1.31 ± 39.64) |  | *TACR1* | Intronic: ENST00000305249, ENST00000409848 |
| rs183242017 | 75,301,987 | G | T | 0.98 | 0.02 | 0.002 | 0.02 | 7.22 (1.31 ± 39.64) |  | *TACR1* | Intronic: ENST00000305249, ENST00000409848 |
| rs187828673 | 75,302,459 | C | T | 0.99 | 0.02 | 0.002 | 0.02 | 7.31 (1.33 ± 40.13) |  | *TACR1* | Intronic: ENST00000305249, ENST00000409848 |
| rs75027902 | 75,303,214 | T | G | 0.99 | 0.02 | 0.002 | 0.02 | 7.31 (1.33 ± 40.13) |  | *TACR1* | Regulatory region: ENSR00001543766; Intronic: ENST00000305249/409848 |
| rs140745583 | 75,304,363 | C | G | 0.96 | 0.03 | 0.004 | 0.02 | 6.97 (1.26 ± 38.45) |  | *TACR1* | Intronic: ENST00000305249, ENST00000409848 |
| rs146658999 | 75,305,009 | C | G | 0.99 | 0.02 | 0.002 | 0.02 | 7.31 (1.33 ± 40.13) |  | *TACR1* | Intronic: ENST00000305249, ENST00000409848 |
| rs190234796 | 75,305,385 | C | A | 0.99 | 0.02 | 0.002 | 0.02 | 7.31 (1.33 ± 40.13) |  | *TACR1* | Intronic: ENST00000305249, ENST00000409848 |
| rs148585513 | 75,306,668 | A | G | 0.99 | 0.02 | 0.002 | 0.02 | 7.31 (1.33 ± 40.13) |  | *TACR1* | Intronic: ENST00000305249, ENST00000409848 |
| rs3771810 | 75,307,653 | T | C | 0.96 | 0.03 | 0.004 | 0.02 | 7.07 (1.28 ± 39) |  | *TACR1* | Intronic: ENST00000305249, ENST00000409848 |
| rs186598817 | 75,312,411 | C | T | 0.98 | 0.02 | 0.002 | 0.02 | 7.15 (1.3 ± 39.27) |  | *TACR1* | Intronic: ENST00000305249, ENST00000409848 |
| rs113546324 | 75,313,527 | A | C | 0.96 | 0.03 | 0.004 | 0.02 | 7 (1.27 ± 38.62) |  | *MIR5000* | Upstream: ENST00000577717; Intronic: ENST00000305249, ENST00000409848 |
| rs189056098 | 75,313,650 | G | A | 0.99 | 0.02 | 0.002 | 0.02 | 7.27 (1.32 ± 39.94) |  | *MIR5000* | Upstream: ENST00000577717; Intronic: ENST00000305249, ENST00000409848 |
| **rs ID**^1^ | **Position Chr2**^2^ | **Imputed SNPs**^3^ | | | | | | |  | **Variant Effect Predictor**^11^ | |
|  |  | **A1**^4^ | **A2**^5^ | **AMPC**^6^ | **F_A**^7^ | **F_U**^8^ | **P**^9^ | **OR (95 % CI)**^10^ |  | **Gene**^12^ | **Consequence of variant**^13^ |
| rs115676714 | 75,315,840 | G | T | 0.96 | 0.05 | 0.004 | 0.02 | 11.36 (1.16 ± 111.15) |  | *MIR5000* | Upstream: ENST00000577717; Intronic: ENST00000305249, ENST00000409848 |
| rs111695084 | 75,316,633 | C | G | 0.96 | 0.05 | 0.005 | 0.02 | 10.77 (1.1 ± 105.4) |  | *MIR5000* | Upstream: ENST00000577717; Intronic: ENST00000305249, ENST00000409848 |
| rs182812841 | 75,316,817 | C | T | 0.96 | 0.05 | 0.005 | 0.03 | 10.48 (1.07 ± 102.52) |  | *TACR1* | Upstream: ENST00000577717; Intronic: ENST00000305249, ENST00000409848 |
| rs189985009 | 75,324,929 | A | G | 0.98 | 0 | 0.04 | 0.01 | -1 |  | *TACR1* | Intronic: ENST00000305249, ENST00000409848 |
| rs148426482 | 75,344,022 | G | A | 0.98 | 0 | 0.03 | 0.03 | -1 |  | *TACR1* | Downstream: ENST00000497764; Intronic: ENST00000305249, ENST00000409848 |
| rs190223821 | 75,347,484 | T | G | 0.98 | 0 | 0.04 | 0.02 | -1 |  | *TACR1* | Intronic: ENST00000497764, ENST00000305249, ENST00000409848 |
| rs3771829 | 75,364,145 | C | G | 1.00 | 0.11 | 0.06 | 0.01 | 1.86 (1.17 ± 2.95) |  | *TACR1* | Intronic: ENST00000305249, ENST00000409848 |
| rs10172990 | 75,364,507 | C | T | 0.98 | 0 | 0.03 | 0.01 | -1 |  | *TACR1* | Intronic: ENST00000305249, ENST00000409848 |
| rs192842575 | 75,365,142 | C | T | 0.98 | 0 | 0.03 | 0.01 | -1 |  | *TACR1* | Intronic: ENST00000305249, ENST00000409848 |
| rs150068826 | 75,366,869 | G | A | 0.98 | 0 | 0.03 | 0.01 | -1 |  | *TACR1* | Intronic: ENST00000305249, ENST00000409848 |
| rs192905448 | 75,366,948 | G | A | 0.97 | 0 | 0.04 | 0.01 | -1 |  | *TACR1* | Intronic: ENST00000305249, ENST00000409848 |
| rs73935568 | 75,368,004 | T | G | 0.97 | 0 | 0.04 | 0.01 | -1 |  | *TACR1* | Intronic: ENST00000305249, ENST00000409848 |
| rs56207850 | 75,368,073 | G | A | 0.97 | 0 | 0.04 | 0.01 | -1 |  | *TACR1* | Intronic: ENST00000305249, ENST00000409848 |
| rs13031966 | 75,368,172 | G | T | 0.97 | 0 | 0.04 | 0.01 | -1 |  | *TACR1* | Intronic: ENST00000305249, ENST00000409848 |
| rs181226824 | 75,368,451 | A | G | 0.97 | 0 | 0.04 | 0.01 | -1 |  | *TACR1* | Intronic: ENST00000305249, ENST00000409848 |
| rs975664 | 75,368,797 | C | T | 0.97 | 0 | 0.04 | 0.01 | -1 |  | *TACR1* | Intronic: ENST00000305249, ENST00000409848 |
| rs180703630 | 75,369,290 | C | T | 0.97 | 0 | 0.04 | 0.01 | -1 |  | *TACR1* | Intronic: ENST00000305249, ENST00000409848 |
| rs9808455 | 75,369,569 | T | C | 0.97 | 0 | 0.04 | 0.01 | -1 |  | - | Regulatory region: ENSR00001543771; Intronic: ENST00000305249/409848 |
| rs193239642 | 75,372,520 | G | C | 0.97 | 0 | 0.04 | 0.01 | -1 |  | *TACR1* | Intronic: ENST00000305249, ENST00000409848 |
| rs58913381 | 75,373,021 | A | G | 0.97 | 0 | 0.04 | 0.01 | -1 |  | *TACR1* | Intronic: ENST00000305249, ENST00000409848 |
| rs143030122 | 75,373,347 | C | T | 0.97 | 0 | 0.04 | 0.01 | -1 |  | *TACR1* | Intronic: ENST00000305249, ENST00000409848 |
| rs145431427 | 75,373,540 | T | C | 0.97 | 0 | 0.04 | 0.009 | -1 |  | *TACR1* | Intronic: ENST00000305249, ENST00000409848 |
| rs57624612 | 75,373,752 | C | T | 0.97 | 0 | 0.04 | 0.009 | -1 |  | *TACR1* | Intronic: ENST00000305249, ENST00000409848 |
| rs191788917 | 75,373,794 | T | A | 0.97 | 0 | 0.04 | 0.01 | -1 |  | *TACR1* | Intronic: ENST00000305249, ENST00000409848 |
| rs12475818 | 75,374,265 | T | G | 0.97 | 0 | 0.04 | 0.01 | -1 |  | - | Regulatory region: ENSR00001543772; Intronic: ENST00000305249/409848 |
| rs192132086 | 75,375,142 | G | C | 0.97 | 0 | 0.04 | 0.008 | -1 |  | *TACR1* | Intronic: ENST00000305249, ENST00000409848 |
| rs184025232 | 75,375,188 | A | G | 0.97 | 0 | 0.04 | 0.007 | -1 |  | *TACR1* | Intronic: ENST00000305249, ENST00000409848 |
| rs72920637 | 75,379,395 | C | T | 0.98 | 0 | 0.04 | 0.01 | -1 |  | *TACR1* | Intronic: ENST00000305249, ENST00000409848 |
| rs192237894 | 75,395,717 | C | T | 0.96 | 0.03 | 0.12 | 0.03 | 0.24 (0.06 ± 1.04) |  | *TACR1* | Intronic: ENST00000305249, ENST00000409848 |
| rs139759288 | 75,398,465 | C | A | 0.96 | 0.008 | 0.05 | 0.03 | 0.16 (0.02 ± 1.22) |  | *TACR1* | Intronic: ENST00000305249, ENST00000409848 |
| rs144204511 | 75,398,919 | G | A | 0.96 | 0.008 | 0.04 | 0.03 | 0.18 (0.02 ± 1.36) |  | *TACR1* | Intronic: ENST00000305249, ENST00000409848 |
| rs2058861 | 75,399,591 | C | T | 0.96 | 0.007 | 0.04 | 0.02 | 0.16 (0.02 ± 1.22) |  | *TACR1* | Intronic: ENST00000305249, ENST00000409848 |
| rs77752293 | 75,401,083 | A | G | 0.96 | 0.29 | 0.45 | 0.001 | 0.49 (0.31 ± 0.78) |  | - | Regulatory region:ENSR00000593128; Intronic: ENST00000305249/409848 |
| rs66833532 | 75,401,540 | G | A | 0.98 | 0.39 | 0.50 | 0.009 | 0.64 (0.44 ± 0.92) |  | *TACR1* | Intronic: ENST00000305249, ENST00000409848 |
| rs72920666 | 75,401,588 | G | A | 0.95 | 0.007 | 0.05 | 0.007 | 0.13 (0.02 ± 0.98) |  | *TACR1* | Intronic: ENST00000305249, ENST00000409848 |
| rs10203484 | 75,401,738 | A | G | 1.00 | 0.44 | 0.34 | 0.003 | 0.67 (0.51 ± 0.87) |  | *TACR1* | Intronic: ENST00000305249, ENST00000409848 |
| rs114917027 | 75,401,923 | T | C | 0.98 | 0.37 | 0.46 | 0.03 | 0.68 (0.46 ± 1) |  | *TACR1* | Intronic: ENST00000305249, ENST00000409848 |
| rs12477554 | 75,402,065 | A | G | 1.00 | 0.50 | 0.42 | 0.02 | 0.72 (0.55 ± 0.94) |  | *TACR1* | Intronic: ENST00000305249, ENST00000409848 |
| rs12469836 | 75,402,895 | C | T | 0.97 | 0.005 | 0.03 | 0.01 | 0.15 (0.02 ± 1.1) |  | *TACR1* | Intronic: ENST00000305249, ENST00000409848 |
| rs13009811 | 75,442,851 | G | A | 0.98 | 0.02 | 0.05 | 0.05 | 0.29 (0.07 ± 1.23) |  | - | Intergenic |
| rs190194915 | 75,442,949 | G | A | 0.97 | 0.007 | 0.05 | 0.02 | 0.16 (0.02 ± 1.16) |  | - | Intergenic |
| rs186770770 | 75,442,994 | G | T | 0.97 | 0.008 | 0.05 | 0.007 | 0.13 (0.02 ± 1) |  | - | Intergenic |
| **rs ID**^1^ | **Position Chr2**^2^ | **Imputed SNPs**^3^ | | | | | | |  | **Variant Effect Predictor**^11^ | |
|  |  | **A1**^4^ | **A2**^5^ | **AMPC**^6^ | **F_A**^7^ | **F_U**^8^ | **P**^9^ | **OR (95 % CI)**^10^ |  | **Gene**^12^ | **Consequence of variant**^13^ |
| rs112638930 | 75,443,694 | T | A | 0.96 | 0.01 | 0.09 | 0.02 | 0.16 (0.02 ± 1.2) |  | - | Intergenic |
| rs1015476 | 75,448,399 | C | T | 0.96 | 0.007 | 0.04 | 0.02 | 0.16 (0.02 ± 1.22) |  | - | Intergenic |
| rs79249323 | 75,469,657 | T | C | 0.96 | 0 | 0.05 | 0.03 | -1 |  | - | Intergenic |
| rs138761610 | 75,483,698 | T | G | 0.95 | 0 | 0.08 | 0.006 | -1 |  | - | Regulatory region: ENSR00001045342; Intergenic |
| rs187628065 | 75,512,594 | G | A | 0.99 | 0.21 | 0.27 | 0.05 | 1.44 (1 ± 2.08) |  | - | Intergenic |
| rs147583893 | 75,522,176 | A | G | 0.98 | 0 | 0.02 | 0.05 | -1 |  | - | Intergenic |
| rs188598766 | 75,527,293 | A | G | 0.96 | 0.17 | 0.26 | 0.05 | 1.69 (1.02 ± 2.77) |  | - | Intergenic |
| rs188844844 | 75,531,543 | C | A | 0.96 | 0.10 | 0.19 | 0.03 | 2.05 (1.08 ± 3.89) |  | - | Intergenic |
| rs189516822 | 75,535,335 | T | C | 0.96 | 0.16 | 0.26 | 0.03 | 1.8 (1.08 ± 3.02) |  | - | Intergenic |
| rs182963771 | 75,538,432 | T | G | 0.96 | 0.10 | 0.18 | 0.02 | 2.1 (1.11 ± 3.98) |  | - | Intergenic |
| rs11695204 | 75,542,192 | T | G | 0.96 | 0.16 | 0.25 | 0.05 | 1.69 (1.03 ± 2.77) |  | - | Intergenic |
| rs181877481 | 75,547,960 | T | C | 0.95 | 0.13 | 0.24 | 0.01 | 2.02 (1.16 ± 3.5) |  | - | Regulatory region: ENSR00001543782; Intergenic |
| rs115347197 | 75,554,080 | G | A | 0.96 | 0.13 | 0.21 | 0.04 | 1.86 (1.04 ± 3.33) |  | - | Intergenic |
| rs35609060 | 75,556,291 | C | G | 0.99 | 0.01 | 0.001 | 0.03 | 10.28 (1.06 ± 99.37) |  | - | Intergenic |
| rs6546969 | 75,556,318 | T | C | 0.99 | 0.01 | 0.001 | 0.03 | 10.28 (1.06 ± 99.37) |  | - | Intergenic |
| rs80176555 | 75,556,325 | G | A | 0.99 | 0.01 | 0.001 | 0.03 | 10.31 (1.07 ± 99.64) |  | - | Intergenic |
| rs9751298 | 75,559,406 | T | A | 0.99 | 0.01 | 0.001 | 0.03 | 10.41 (1.08 ± 100.59) |  | - | Intergenic |
| rs183653669 | 75,585,445 | C | T | 0.95 | 0.10 | 0.28 | 0.01 | 3.64 (1.24 ± 10.68) |  | - | Intergenic |
| rs142206799 | 75,587,362 | A | C | 0.95 | 0.09 | 0.27 | 0.006 | 3.97 (1.36 ± 11.59) |  | - | Intergenic |
| rs181128842 | 75,588,551 | G | A | 0.95 | 0.13 | 0.26 | 0.04 | 2.52 (1.01 ± 6.25) |  | - | Intergenic |
| rs186597826 | 75,591,991 | C | A | 0.99 | 0.07 | 0.14 | 0.007 | 2.22 (1.18 ± 4.15) |  | - | Intergenic |
| rs138892253 | 75,593,975 | T | A | 0.99 | 0.10 | 0.16 | 0.05 | 1.67 (0.98 ± 2.85) |  | - | Intergenic |
| rs147375239 | 75,594,868 | C | T | 0.99 | 0.10 | 0.16 | 0.05 | 1.67 (0.98 ± 2.85) |  | - | Intergenic |
| rs188412181 | 75,597,950 | T | C | 0.99 | 0.10 | 0.16 | 0.04 | 1.75 (1.01 ± 3.01) |  | - | Intergenic |
| rs182585347 | 75,598,910 | C | T | 0.97 | 0.09 | 0.16 | 0.03 | 1.97 (1.06 ± 3.64) |  | - | Intergenic |
| rs55892887 | 75,603,302 | C | A | 0.98 | 0.06 | 0.13 | 0.01 | 2.18 (1.13 ± 4.19) |  | - | Intergenic |
| chr2:75604406:I | 75,604,406 | T | TA | 0.98 | 0.06 | 0.12 | 0.02 | 2.12 (1.06 ± 4.22) |  | - | Intergenic |
| rs186436818 | 75,604,808 | G | T | 0.98 | 0.06 | 0.12 | 0.02 | 2.19 (1.1 ± 4.35) |  | - | Intergenic |
| rs141585722 | 75,605,638 | A | G | 0.98 | 0.06 | 0.13 | 0.009 | 2.39 (1.17 ± 4.91) |  | - | Intergenic |
| rs78795632 | 75,606,177 | T | A | 0.98 | 0.06 | 0.12 | 0.03 | 2.14 (1.04 ± 4.42) |  | - | Intergenic |
| rs182831926 | 75,607,123 | G | A | 0.99 | 0.04 | 0.11 | 0.008 | 2.69 (1.2 ± 6) |  | - | Regulatory region: ENSR00000593145; Intergenic |
| rs79502391 | 75,610,667 | G | T | 0.98 | 0.05 | 0.10 | 0.03 | 2.33 (1.04 ± 5.25) |  | - | Intergenic |
| rs13412796 | 75,617,057 | C | T | 0.95 | 0 | 0.05 | 0.02 | -1 |  | - | Intergenic |
| rs75210098 | 75,618,847 | C | G | 0.94 | 0 | 0.05 | 0.04 | -1 |  | - | Intergenic |
| rs186147120 | 75,619,375 | C | T | 0.97 | 0 | 0.03 | 0.002 | -1 |  | - | Intergenic |
| rs184286063 | 75,628,209 | T | C | 0.99 | 0 | 0.04 | 0.01 | -1 |  | - | Intergenic |
| rs7422327 | 75,629,867 | A | C | 0.99 | 0.03 | 0.11 | 0.005 | 0.22 (0.07 ± 0.71) |  | - | Intergenic |
| rs1160613 | 75,632,552 | G | T | 1.00 | 0.04 | 0.01 | 0.02 | 2.8 (1.22 ± 6.46) |  | - | Intergenic |
| rs7567151 | 75,633,124 | C | A | 1.00 | 0.04 | 0.01 | 0.02 | 2.77 (1.2 ± 6.38) |  | - | Intergenic |
| rs10185346 | 75,681,696 | A | G | 0.99 | 0.02 | 0.09 | 0.01 | 0.23 (0.05 ± 0.97) |  | *GAPDHP57* | Downstream: ENST00000424400 |
| rs148204204 | 75,689,855 | G | C | 1.00 | 0.02 | 0.15 | 0.0003 | 0.14 (0.03 ± 0.57) |  | *-* | Intergenic |
| rs188247076 | 75,691,402 | G | T | 1.00 | 0.02 | 0.15 | 0.0003 | 0.14 (0.03 ± 0.57) |  | *-* | Intergenic |
| **rs ID**^1^ | **Position Chr2**^2^ | **Imputed SNPs**^3^ | | | | | | |  | **Variant Effect Predictor**^11^ | |
|  |  | **A1**^4^ | **A2**^5^ | **AMPC**^6^ | **F_A**^7^ | **F_U**^8^ | **P**^9^ | **OR (95 % CI)**^10^ |  | **Gene**^12^ | **Consequence of variant**^13^ |
| rs191379486 | 75,692,291 | G | A | 0.99 | 0.03 | 0.12 | 0.0009 | 0.2 (0.06 ± 0.65) |  | *FAM176A* | Downstream: ENST00000485891, ENST00000490746 |
| rs17011370 | 75,692,623 | C | T | 1.00 | 0.02 | 0.07 | 0.0001 | 0.21 (0.08 ± 0.58) |  | *-* | Regulatory region: ENSR00001543786; Downstream: ENST00000485891/490746 |
| chr2:75693696:D | 75,693,696 | AC | A | 0.99 | 0.03 | 0.11 | 0.001 | 0.21 (0.06 ± 0.69) |  | *FAM176A* | Downstream: ENST00000485891, ENST00000490746 |
| rs150650642 | 75,694,706 | C | T | 0.99 | 0.03 | 0.11 | 0.001 | 0.21 (0.06 ± 0.69) |  | *FAM176A* | Downstream: ENST00000485891, ENST00000490746 |
| rs147876791 | 75,695,610 | C | A | 0.97 | 0 | 0.02 | 0.007 | -1 |  | *FAM176A* | Downstream: ENST00000485891, ENST00000490746 |
| rs187483169 | 75,695,718 | G | T | 0.97 | 0.01 | 0.05 | 0.01 | 0.22 (0.05 ± 0.94) |  | *FAM176A* | Downstream: ENST00000485891, ENST00000490746 |
| rs72814774 | 75,696,529 | T | A | 1.00 | 0 | 0.009 | 0.04 | -1 |  | *FAM176A* | Intronic: ENST00000485891, ENST00000490746 |
| rs144915431 | 75,698,279 | G | C | 0.97 | 0.004 | 0.04 | 0.001 | 0.11 (0.01 ± 0.78) |  | *FAM176A* | Upstream: ENST00000444852; Intronic: ENST00000485891, ENST00000490746 |
| rs4852379 | 75,698,352 | A | G | 1.00 | 0 | 0.009 | 0.04 | -1 |  | *FAM176A* | Upstream: ENST00000444852; Intronic: ENST00000485891, ENST00000490747 |
| rs6756748 | 75,699,657 | C | T | 1.00 | 0.03 | 0.14 | 0.002 | 0.21 (0.06 ± 0.7) |  | *FAM176A* | Upstream: ENST00000444852; Intronic: ENST00000485891, ENST00000490748 |
| rs139919044 | 75,699,744 | C | T | 0.97 | 0 | 0.04 | 0.001 | 0.11 (0.01 ± 0.78) |  | *FAM176A* | Upstream: ENST00000444852; Intronic: ENST00000485891, ENST00000490749 |
| rs190745719 | 75,699,981 | C | T | 0.98 | 0.03 | 0.11 | 0.001 | 0.21 (0.06 ± 0.68) |  | *FAM176A* | Upstream: ENST00000444852; Intronic: ENST00000485891, ENST00000490750 |

^1^ Reference SNP, single nucleotide polymorphism, (rs) ID given; I, insertion, D, deletion.

^2^ Position on Chromosome 2, hg19 Build NCBI37, May 2009.

^3^ Imputed SNPs, using IMPUTE2 software to generate tests of association (Howie et al., 2009; Howie et al., 2011).

^4^ A1, First Allele.

^5^ A2, Second Allele.

^6^ AMPC, average maximum posterior call, which is the probability across all individuals in the sample that are used for the test at each SNP. This is a measure of how much uncertainty there is at each SNP. Samples excluded will be (a) those excluded using the -exclude_samples option, (b) samples with a missing phenotype or covariate relevant to the test, (c) samples without genotypes if the -method threshold option is used, (d) samples where the sum of the genotype probabilities is less than 0.1 (Marchini and Howie 2010).

^7^ F_A, Allele frequency in the affected individuals

^8^ F_U, Allele frequency in the unaffected individuals

^9^ P value, Significance value of case/control association test using the phenotype frequentist additive model P value (≤ 0.05) tested at each SNP versus a model of no association. The beta estimates of the additive model increase in log-odds that can be attributed to each copy of allele 2. When a model cannot be fitted to the data the p-value is set to -1 (Marchini and Howie 2010).

^10^ OR (95 % CI), odds ratio with 95 % confidence intervals in parentheses; -1: metric was not calculated by the IMPUTE2 software.

^11^ Variant Effect Predictor, server used to identify the functional effects of each significantly associated imputed SNP (McLaren 2010).

^12^ Gene, *TACR1* Ensembl ID ENSG00000115353; *GAPDHP57* Ensembl ID ENSG00000236167, a non-codig processed pseudogene; *FAM176A* Ensembl ID ENSG00000115363, *Homo sapiens* family with sequence similarity 176, member A.

^13^ Consequence of variant, notes about where and in which Ensembl transcript (ENST) or Ensembl regulatory region (ENSR) each imputed SNP variant is located. All intergenic and intronic regulatory region variants are located in regions which show enrichment of the H3K27Ac histone mark, which is the acetylation of lysine 27 of the H3 histone protein, according to the ENCODE regulation supertrack on UCSC genome browser (ENCODE Project Consortium 2011).
